# Prognostic and Spatial Organization of Tertiary Lymphoid Structures in Pancreatic Ductal Adenocarcinoma

**DOI:** 10.3390/biomedicines14102287

**Published:** 2026-10-09

**Authors:** Yunqi Jia, Mengzhe Zhang, Haoran Yang, Yuhan Shi, Shiyuan Zhang

**Affiliations:** 1 First Teaching Hospital of Tianjin University of Traditional Chinese Medicine, National Clinical Research Center for Chinese Medicine, Tianjin 300193, China; nancyjia96@163.com; 2 Department of Thoracic Surgical Oncology, Shanghai Chest Hospital, Shanghai Institute of Thoracic Oncology, Shanghai Jiao Tong University School of Medicine, Shanghai 200030, China; zhangmengzhe1996@tmu.edu.cn; 3 Department of Lung Cancer Surgery, Tianjin Medical University Cancer Institute and Hospital, National Clinical Research Center for Cancer, Key Laboratory of Cancer Prevention and Therapy, Tianjin’s Clinical Research Center for Cancer, Tianjin Lung Cancer Center, Tianjin 300060, China; 4 Tianjin Medical University Cancer Institute and Hospital, Huan-Hu-Xi Road, Ti-Yuan-Bei, Hexi District, Tianjin 300060, China; yang-haoran@tmu.edu.cn; 5 The Second Hospital of Tianjin Medical University, 23 Pingjiang Road, Hexi District, Tianjin 300211, China; shiyuhan0504@tmu.edu.cn

**Keywords:** pancreatic ductal adenocarcinoma, tertiary lymphoid structures, prognostic biomarker, spatial transcriptomics, tumor microenvironment, cancer-associated fibroblasts

## Abstract

**Background**: Tertiary lymphoid structures (TLSs) may identify immunologically active pancreatic ductal adenocarcinoma (PDAC), but clinical definitions and spatial analyses have been inconsistent. **Methods**: We integrated a systematic review of 21 studies (22 reports) with patient-aware secondary analyses of seven public molecular datasets. **Results**: Pathology-defined TLS presence was associated with longer overall survival in five cohorts (1019 patients; random-effects hazard ratio 0.544, 95% confidence interval 0.398–0.743). In pathology-annotated spatial data, a mature-TLS program was enriched in TLS-positive regions at the patient level. B-cell and Tfh/CXCL13 programs showed reproducible positive associations with continuous TLS burden, whereas other program associations were less directionally consistent. An independent spatial cohort supported an rCAF-over-myCAF association with lymphoid probability. Chemokine-axis co-enrichment was observed but did not establish direct signaling. **Conclusions**: TLSs mark a favorable, spatially organized immune state in PDAC. The evidence supports biomarker and biological hypotheses while retaining boundaries on causality, treatment induction, and clinical prediction.

## 1. Introduction

Pancreatic ductal adenocarcinoma (PDAC) remains among the most lethal solid malignancies. Its desmoplastic stroma, dysfunctional myeloid compartment, sparse effector lymphocyte infiltration, and extensive inter- and intratumoral heterogeneity limit the efficacy of immunotherapy. Nevertheless, some PDACs contain tertiary lymphoid structures (TLSs), organized immune niches that may support local antigen presentation and lymphocyte maturation [1,2].

For pancreatic cancer overall, the 5-year relative survival was 13.7%, with a marked stage gradient from 43.6% for localized disease to 3.4% for distant disease [3]. These population-level estimates provide clinical context for evaluating prognostic features such as TLSs, but they should not be interpreted as survival estimates for the resected cohorts analyzed here.

The meaning of a TLS depends on its maturity, location, and stromal context rather than on a binary presence/absence label. In PDAC, inflammatory, myofibroblastic, and antigen-presenting cancer-associated fibroblast (CAF) programs occupy distinct microanatomic niches [4,5,6]. An emerging FAP-positive CAF state and stromal-intervention studies further support state-specific, rather than pan-stromal, interpretation [7,8,9]. This caution is also supported by stromal reprogramming work [10].

Genomic and single-cell studies have resolved PDAC into molecular and microenvironmental states that are not captured by tumor cellularity alone [11,12,13]. Early pathology studies linked intratumoral or peritumoral lymphoid organization to favorable outcome [14,15], whereas germinal-center activity and vaccine-associated lymphoid aggregates have been related to long-term survivorship and immune activation [16,17]. More recent reports emphasize heterogeneity in TLS location, abundance, maturation, and treatment context [18,19,20].

Clinical synthesis is complicated by incompatible TLS constructs, including presence, density, area, location, maturity, and marker-defined lymphoid aggregates. Spatial and single-cell studies pose a second problem: cells, spots, and regions are repeated measurements within a small number of patients. This study therefore treats the patient or specimen, rather than the molecular measurement, as the unit of inference whenever possible.

We integrated a systematic clinical evidence synthesis with patient-aware reanalysis of public molecular datasets. GSE202051 maps treatment-associated cellular remodeling [21]; GSE277116 couples spatial transcriptomics with pathology-derived TLS annotations [22]; and GSE322767 provides an independent spatial test of CAF programs in relation to lymphoid probability [23]. Single-cell processing used established Seurat workflows [24,25]. The corresponding deconvolution and biological-replication methods are described and referenced in the Methods. We asked whether mature-TLS, B-cell, Tfh/CXCL13, FDC/HEV, and fibroblast programs show reproducible patient-level associations, while retaining explicit boundaries on causality and treatment effects.

## 2. Materials and Methods

The analysis combined a systematic review with secondary reanalysis of public molecular datasets. Database searches covered records through 25 July 2026. The clinical systematic review was registered in PROSPERO (CRD420261465162) on 30 July 2026, after searching, initial screening, and preliminary quantitative analysis had begun. For the harmonized secondary molecular reanalysis, we used an internally defined hypothesis-guided framework comprising six biological questions: (H1) mature/invasive-border TLSs have higher mature-TLS scores; (H2) GC B, Tfh/CXCL13, and FDC/HEV programs form a reproducible maturation axis; (H3) rCAF organizer programs align with TLS whereas myCAF/TGFβ programs align with TLS resistance; (H4) treatment context associates with patient- or specimen-level immune and stromal remodeling; (H5) CXCL13–CXCR5, CCL19/21–CCR7, and LTB–LTBR receive repeated spatial support; and (H6) long-term recurrence-free patients have higher mature-TLS scores. These hypotheses and program definitions were not prospectively registered before inspection of all contributing molecular datasets and are therefore not treated as prospectively prespecified confirmatory hypotheses. The shared analysis configuration fixes the operational gene-program and chemokine-axis definitions used for the harmonized reanalysis. The dated GSE202051 decision record of 16 August 2026 documents the GSE202051 12-population composition family/model and the 50-cell compartment eligibility threshold before those models were fitted. The eight-specimens-per-group rule is documented in the retained scoring script, not the above-mentioned dated record. The record does not establish prespecification of the other datasets. Patient or specimen was the inferential unit whenever donor identity was recoverable. Biological rationale, dataset mapping, and interpretation boundaries are reported in Supplementary Method S3.

### 2.1. Systematic Search and Study Selection

Database searches covered records from database inception through 25 July 2026 in PubMed/MEDLINE, Embase, Scopus, Web of Science Core Collection, and Wanfang. The search combined controlled vocabulary and free-text terms for PDAC and tertiary lymphoid structures, without an outcome filter. No language or publication-type restriction was applied at the search stage. Reference lists of included reports and relevant reviews were examined, and forward citation tracking was used to identify additional records. Records were deduplicated using DOI, PMID, title, author, and year, followed by manual verification. No formal gray-literature search was conducted; gray-literature databases, preprint servers, conference-proceeding databases, and trial registries were not searched. Title/abstract screening and full-text assessment were performed independently by two reviewers; disagreements were resolved by discussion, with third-reviewer adjudication when required.

Reporting followed PRISMA 2020 and the PRISMA-S search-reporting extension; prognostic-study bias was structured with QUIPS [26,27,28].

Eligible studies included adults with histologically confirmed PDAC and a direct tissue-based TLS measure defined by histology, immunohistochemistry, multiplex imaging, or pathology-validated image analysis. Pure expression-signature studies without pathology anchoring were excluded from the primary clinical meta-analysis. The primary endpoint was overall survival. Cause-specific, disease-specific, disease-free, recurrence-free, and progression-free survival were retained as distinct secondary endpoints. Upfront and post-neoadjuvant cohorts were not pooled across treatment context. The independent patient cohort, rather than publication, was the unit of clinical synthesis.

### 2.2. Clinical Data Extraction and Effect Harmonization

Two reviewers independently extracted study design, recruitment setting, treatment context, cohort size, tissue sampling, TLS definition, anatomical location, maturation criteria, positivity threshold, endpoint definition, follow-up, effect estimate, adjustment variables, and potential cohort overlap into structured forms. They independently checked every effect included in a quantitative synthesis against the source report. Disagreements were resolved by discussion, with third-reviewer adjudication when consensus could not be reached. Adjusted Cox hazard ratios were preferred. Effects were oriented so that HR < 1 indicated better survival in the group with TLSs present, and being more abundant, more organized, more mature, or located at a prespecified favorable site. Estimates reported with the opposite reference category were inverted together with their confidence limits. Reports from overlapping cohorts were linked, and only one estimate from a participant set was retained within any analysis pool. Clinical cohort characteristics and extraction status are provided in Table S1, and harmonized clinical association effects and source data are provided in Table S2.

### 2.3. Clinical Meta-Analysis

The primary OS synthesis was restricted to five independent resection cohorts reporting adjusted Cox estimates for a direct binary, pathology-defined TLS-presence contrast. Three cohorts defined intratumoral TLS/TLO presence and two defined any-site TLS presence. These constructs were combined because each directly contrasted histological presence with absence in resected PDAC and used the same survival direction; density-, maturity-, continuous-area-, expression-signature-, and post-neoadjuvant effects were excluded. A location-restricted intratumoral analysis was reported separately. Log HRs were synthesized using generic inverse-variance random-effects models. Between-study variance was estimated by restricted maximum likelihood, Hartung–Knapp inference was used for confidence intervals, and prediction intervals were calculated for pools with at least three cohorts using the variance-based method. Prevalence was modeled using a random-intercept logistic model with maximum-likelihood between-study variance and logit transformation; study-specific proportions used exact binomial confidence intervals. Post-neoadjuvant prevalence was not pooled because only two clinically incompatible cohorts were available. Heterogeneity was summarized using τ^2^, I^2^, Cochran’s Q, and prediction intervals. Funnel-plot asymmetry tests; Egger tests; and meta-regression, trim-and-fill, and p-curve analyses were not performed because every synthesis contained fewer than ten studies. DFS, RFS, and PFS were preserved as distinct outcomes; the additional cross-endpoint synthesis was post hoc and exploratory and was used only to assess direction.

Random-effects modeling and Hartung–Knapp–Sidik–Jonkman inference followed established small-meta-analysis guidance [29,30].

### 2.4. Risk of Bias

Two reviewers independently assessed risk of bias with QUIPS across study participation, study attrition, prognostic-factor measurement, outcome measurement, study confounding, and statistical analysis and reporting. Judgments were supported by source-level evidence and resolved by consensus, with third-reviewer adjudication when required. QUIPS domain judgments were reported without imposing a non-standard overall score. A sensitivity analysis excluded reports with at least one high-risk domain. QUIPS was treated as a risk-of-bias framework rather than a certainty-of-evidence assessment.

### 2.5. Fixed Gene-Program Definitions for Harmonized Reanalysis

Whole-transcriptome analyses used the eight TLS gene programs defined in the shared analysis configuration. The configuration provides a fixed implementation for the harmonized reanalysis but is not used as evidence of prospective prespecification. Full program membership, analysis-specific score construction, specimen/compartment eligibility, and dataset-specific availability rules are reported in Supplementary Method S2. For NanoString targeted panels, scoring was limited to genes measured by the platform; platform-specific minimum coverage rules are listed in S2.3. No gene was added in response to an observed association within a given analysis.

Multiple-testing families were controlled with the Benjamini–Hochberg false-discovery-rate procedure [31]; bootstrap uncertainty and exact tests were applied at the independent-unit level [32,33]. The analysis claim-to-estimand and reproducibility-status register is provided in Table S3.

### 2.6. GSE202051 Single-Nucleus Analysis

The full processed AnnData object was audited for file integrity, dimensions, cell annotations, specimen identifiers, treatment labels, and duplicate libraries. Analyses included 224,988 nuclei from 43 PDAC specimens (18 untreated, 25 treated). Cell counts were aggregated by specimen and annotated population. For all 12 Level 1 populations, treated-versus-untreated differences in specimen-level proportions were modeled using equal-specimen-weight quasi-binomial regression. All 12 populations formed a single composition multiplicity family, with BH correction across the 12 tests; this model family was documented before raw-object composition-model fitting in the dated decision record. No standard-error-based population exclusion was applied. TLS-program analyses used the supplied specimen-by-compartment pseudobulk logCPM table. Within each biologically assigned compartment, gene logCPM values were z-standardized across eligible specimens; zero-variance or non-finite gene scores were omitted, and each program score was the mean of the remaining gene scores. A specimen required at least 50 cells in the relevant compartment, and each treatment-group comparison required at least eight eligible specimens per group. The 50-cell threshold is documented in the dated GSE202051 decision record; the minimum of eight specimens per group is implemented in the retained scoring script. The five lymphoid programs were evaluated in the Lymphoid compartment, FDC/HEV in Endothelial, and rCAF-organizer and myCAF/TGFβ in Cancer-associated fibroblast. Equal-specimen-weight linear models estimated treated-minus-untreated program-score contrasts, with BH correction jointly across all eight program tests. The supplied logCPM values were not treated as raw counts for edgeR/DESeq2 inference.

Single-cell processing and integration used established Seurat workflows [24,25], with versions and session information recorded for transparent handling.

### 2.7. GSE277116 Spatial TLS Analysis

Space Ranger matrices, barcodes, tissue coordinates, pathology TLS annotation, and public sample metadata were audited for the 12 CODA-annotated sections. The original study code was used to recover the section-to-patient map: 12 sections represented 10 patients, with two patients contributing both a tumor and a lymph-node section. Spots were categorized as TLS-zero, TLS-positive, and a higher-threshold sensitivity set using the fixed pathology annotation.

Within each section, program effects were calculated as standardized TLS-positive-minus-TLS-zero differences. Sections were first averaged within patient. Patient effects were summarized overall, in tumor specimens, and in lymph-node specimens. Confidence intervals were obtained by patient bootstrap. Exact two-sided sign-flip tests enumerated all 2^*n* patient sign assignments. BH correction was applied across fixed programs within tissue scope. Section-level estimates were shown descriptively and were not treated as independent patients.

Spatial spots, sections, regions, and nuclei were treated as subsampling or observational units rather than independent biological replicates. Patient-level aggregation and exact inference were used whenever donor identity was recoverable to avoid pseudoreplication, consistent with methodological guidance on biological-replicate-aware single-cell inference [34,35].

For continuous ecology analysis, the Spearman correlation between pathology TLS percentage and each program was calculated separately within every tumor patient. For the descriptive summary, patient correlations were Fisher-z transformed, averaged across the eight patients, and back-transformed to rho. Pointwise 95% percentile confidence intervals used 10,000 patient resamples; the mean Fisher-z correlation was calculated in each resample and back-transformed before taking the 2.5th and 97.5th percentiles. The primary exact two-sided sign-flip tests used the raw patient-specific correlations; the exact binomial sign test assessed directional concordance as a sensitivity analysis. BH correction was applied separately to each test type across the same seven programs. The summary table distinguishes the displayed Fisher-z estimate from the arithmetic mean of raw correlations. The confidence intervals are not multiplicity-adjusted.

### 2.8. RCTD Reference Robustness and TLS-Neighborhood Analysis

RCTD multi-mode deconvolution [36] used the donor-balanced GSE202051 reference and all in-tissue spots with at least 100 UMIs. For reference-composition sensitivity, the reference was rebuilt using untreated GSE202051 specimens only; section map, spots, TLS contrasts, patient aggregation, and effect definitions were otherwise fixed. Finite patient-by-cell-type effects from the two reference analyses were compared by Pearson and Spearman correlation and directional agreement.

For the exploratory TLS-neighborhood analysis, a TLS-zero spot was classified as a neighborhood spot if at least one of its six nearest spots by 10x Visium image pixel coordinates (pxl_col_in_fullres and pxl_row_in_fullres) was TLS-positive; the remaining TLS-zero spots formed the background. TLS-positive labels were permuted 999 times within each tumor section while preserving the observed positive-spot count. For each of the 21 cell types meeting the reference-cell count threshold, neighborhood-minus-background RCTD weight effects were aggregated within patient. Two-sided exact patient-level sign-flip tests were BH-corrected across the 21 cell types; section-wise permutation empirical *p* values were also BH-corrected across the same family. This exploratory analysis tests co-localization under a random-label null, not migration, recruitment, or causality.

### 2.9. GSE322767 CAF Spatial Analysis

Eight patient Seurat objects were checked against recorded SHA-256 hashes and audited for counts, coordinates, histology alignment, lymphoid probability, and six deposited CAF scores (apCAF, iCAF, myCAF, myFb.4, myFb.5, and rCAF). No categorical TLS-permissive/TLS-resistant or treatment label was inferred. For each patient and CAF program, Spearman correlation with continuous lymphoid probability was calculated. Patients were the independent units. For the descriptive display, the mean Fisher-z correlation was back-transformed to rho, with pointwise 95% percentile confidence intervals from 10,000 patient resamples calculated on the same scale. The result table separately reports arithmetic_mean_rho for the raw-correlation mean; mean_rho and its interval refer to the displayed Fisher-z summary. Exact two-sided sign-flip tests were enumerated across 2^8 assignments using the raw patient correlations, and BH correction covered all six programs. The exact binomial sign test was a directional sensitivity analysis with its own six-program BH adjustment. Pointwise intervals are not multiplicity-adjusted.

The primary direct stromal contrast subtracted each patient’s myCAF correlation from its rCAF correlation. Exact sign-flip inference was applied to the eight within-patient differences. Robustness was assessed by omitting one patient at a time. A sensitivity analysis compared top and bottom quartiles of lymphoid probability within each patient using standardized mean differences.

### 2.10. Ligand–Receptor Axis Analysis

The hypothesis-guided axes were defined as the mean standardized expression of CXCL13/CXCR5, CCL19/CCL21/CCR7, and LTB/LTBR. Coverage was verified in every GSE277116 section. Primary and sensitivity standardized differences were calculated within section, aggregated within patient, and tested among eight tumor patients by exact sign-flip. BH correction was performed across the three axes and separately across seven component genes. These scores were interpreted as spatial co-enrichment and not as direct cell–cell communication.

### 2.11. Exploratory TLS-Core Distance-Gradient Analysis

For GSE277116 tumor sections, TLS-core spots had a pathology TLS percentage of at least 50. A weighted TLS-core centroid was calculated per section and each spot was assigned to a core, near, mid, or far distance shell. For each fixed axis, the section-level core-minus-far standardized difference was aggregated within patient. Exact two-sided sign-flip tests across eight patients were BH-adjusted across the three axes. This post hoc analysis was explicitly exploratory and was interpreted only as a test of local expression co-enrichment.

### 2.12. Exploratory Cross-Dataset Targeted-Panel Assessments

GSE225767 included 20 post-SBRT PDAC samples (15 responders and 5 non-responders); the eight-gene mature-TLS score was evaluated with an exact two-sided label-permutation test. GSE220929 included 10 long-term recurrence-free and 10 short-term recurrent PDAC tumors. The library-size-normalized targeted-panel mature-TLS score was compared using the long-minus-short mean difference and Hedges’ g. The two-sided permutation *p* value was calculated by enumerating all 184,756 allocations of 10 of the 20 patients to one group and comparing the absolute permuted mean difference with the absolute observed mean difference. GSE240078 contributed patient-aggregated stromal measurements from 40 patients; because only a two-gene B/GC proxy was available, this treated-versus-untreated comparison was designated exploratory. For the three patient-level external cohorts mean differences and 95% percentile confidence intervals were calculated using 7000 patient-level bootstrap resamples for each cohort. For visual comparison across the same three patient-level external contexts, standardized effect sizes (Hedges g) and 95% percentile confidence intervals were likewise calculated using 7000 patient-level bootstrap resamples and displayed side by side; no pooled or meta-analytic estimate was calculated. Fixed random seeds and the patient-level source tables are provided in the reproducibility package. GSE226840 comprised 19 laser-capture microdissections, including 14 PDAC-associated TLS regions classified as CD23-negative, CD23-low, or CD23-high and five adjacent-normal samples. The source documentation states that the TLS microdissections arose from tumors from eight patients, but a region-to-patient mapping sufficient for donor-aware analysis was not recoverable from the public metadata or deposited analytical files used here. Because multiple regions could therefore originate from the same patient, region-level observations were not treated as independent biological replicates and no inferential maturation-trend test was performed. GSE226840 was retained only as a dataset-level audit of gene coverage and TLS maturation categories. It was not used to support or refute cross-dataset replication. The three patient-level inferential external contexts differed in platform, endpoint, and gene coverage and were not pooled.

For the Bayesian sensitivity analysis shown, we used a normal–normal hierarchical model on the log-HR scale: y_i_|θ_i_~Normal(θ_i_, σ_i_^2^) and θ_i_|μ,τ~Normal(μ, τ^2^). The reference model used μ~Normal(0, 2^2^) and τ~half-Normal(0.25). Sensitivity models used τ~half-Normal(0.50), τ~half-Cauchy(0.50), or a skeptical effect prior μ~Normal(0, 0.5^2^) with τ~half-Normal(0.25). Posterior quantities were obtained by deterministic numerical quadrature over τ from 0 to 10 using 24,001 grid points, with μ integrated analytically conditional on τ. Numerical adequacy was checked by successful posterior normalization and relative posterior density below 10^−7^ at the upper τ boundary. No MCMC sampling was used; R-hat and MCMC effective sample-size diagnostics were therefore not applicable. P(HR < 1) denotes posterior probability that the pooled mean association favors TLS presence under the stated data, model, and prior—not a frequentist *p* value. The displayed intervals are central 95% credible intervals, and posterior predictive intervals were retained to show uncertainty for a future setting.

### 2.13. Quantification and Statistical Analysis

All tests were two sided. Effect estimates, confidence intervals, independent sample size, and multiplicity-adjusted *p* values are reported where applicable. Comparisons sharing a dataset, estimand, biological level, and analysis type were grouped into multiple-testing families. BH-FDR was controlled within each stated family, including the 12 GSE202051 composition tests, six GSE322767 CAF programs, three chemokine-axis composites, seven component genes, and 21 RCTD neighborhood cell types. Supplementary Table S4 reports the principal families, their sizes, and the rationale for grouping. The dated pre-model GSE202051 decision record documents the 12-population composition family; the grouping rationale for other analyses does not establish that their families were defined before the contributing datasets were examined. Within-family adjustment does not provide study-wide confirmatory error control. The external-dataset *p* values are cohort-specific and are not corrected across all exploratory questions in this study. Accordingly, secondary, sensitivity, and cross-dataset analyses are interpreted as hypothesis-guided or exploratory; nominal or within-family significance is not treated as independent validation or used to overturn unsupported primary findings.

## 3. Results

### 3.1. Clinical Evidence Establishes a Favorable but Heterogeneous TLS Phenotype

We first performed a systematic review and structured evidence synthesis of pathology-defined TLSs in PDAC. The searches through 25 July 2026 identified 1188 records (PubMed/MEDLINE, 119; Embase, 367; Scopus, 326; Web of Science Core Collection, 215; Wanfang, 161). After removal of 209 duplicates, 979 records underwent title/abstract screening; 62 reports were sought for full-text retrieval, six could not be obtained, and 56 were assessed for eligibility. Twenty-one studies represented by 22 reports were included; 18 reports contributed at least one quantitative estimate and four contributed qualitative or contextual evidence only (Figure S3; Table 1). The evidence architecture was intentionally layered: clinical meta-analysis established the prognostic anchor, single-nucleus data tested treatment-associated remodeling, pathology-anchored spatial data tested TLS organization, an independent spatial cohort tested fibroblast ecology, and targeted panels provided exploratory cross-dataset assessments.

**Figure 1:**
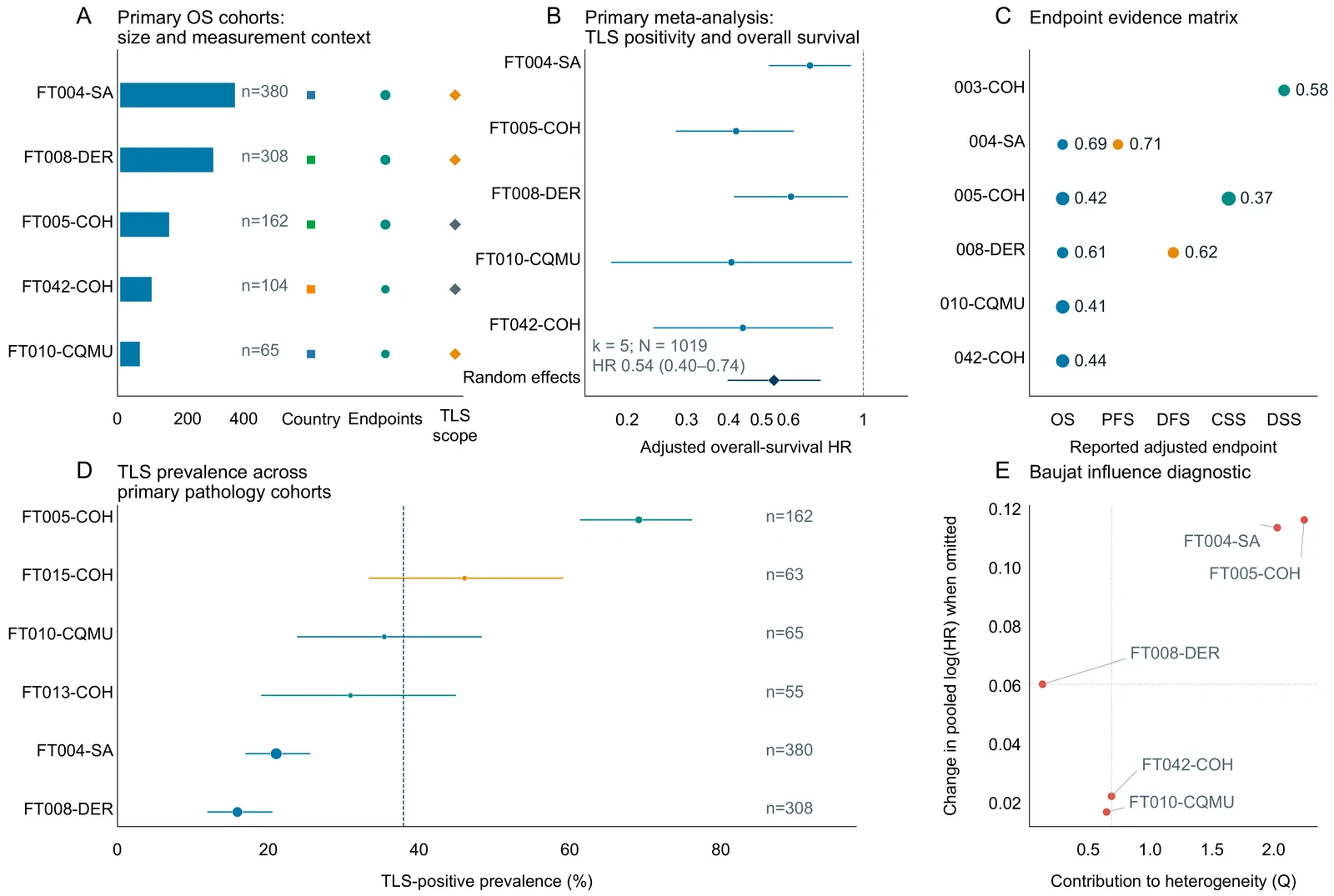
Meta-analytic evidence landscape for pathology-defined tertiary lymphoid structures (TLSs) in pancreatic ductal adenocarcinoma (PDAC). (**A**) Cohort size and measurement context for the five primary overall-survival cohorts. Bars represent cohort size. Country squares are blue for China, green for Japan, and orange for Germany. Teal circles indicate reported endpoint coverage, with larger circles indicating two rather than one reported endpoint. TLS-scope diamonds are orange for an intratumoral definition and gray for a definition not restricted to intratumoral TLSs. (**B**) Adjusted cohort-specific hazard ratios (HRs; circles) and 95% confidence intervals (horizontal lines), with the random-effects summary shown as a diamond. The vertical dashed line denotes HR = 1; the HR axis is logarithmic. The model used restricted maximum likelihood estimation and Hartung–Knapp inference. (**C**) Endpoint evidence matrix. Blue bubbles denote overall survival (OS), orange bubbles denote progression-free survival (PFS) or disease-free survival (DFS), and teal bubbles denote cause-specific survival (CSS) or disease-specific survival (DSS). Bubble area represents |log(HR)|; adjacent numbers report HRs, and blank cells indicate unreported endpoints. (**D**) Study-specific TLS prevalence estimates in the displayed primary pathology cohorts, with 95% confidence intervals. Blue denotes intratumoral TLSs, teal denotes TLS presence without an intratumoral-only restriction, and orange denotes early TLSs. Marker size reflects cohort size, with a minimum size for smaller cohorts. The dashed line marks the pooled prevalence of 37.9%. (**E**) Baujat influence diagnostic. The horizontal axis shows contribution to heterogeneity, and the vertical axis shows the change in pooled log(HR) when a cohort is omitted. Points toward the upper right indicate greater heterogeneity contribution and influence. HRs below 1 favor TLS presence. Cohorts, rather than reports, are the independent units.

Five independent resection cohorts comprising 1019 patients contributed adjusted Cox estimates for a direct binary, pathology-defined TLS-presence contrast. A random-effects model using restricted maximum likelihood and Hartung–Knapp inference yielded a pooled hazard ratio (HR) of 0.544 (95% CI 0.398–0.743; *p* = 0.0056), with moderate estimated heterogeneity (I^2^ = 30.3%) and a prediction interval of 0.304–0.973 (Figure 1A,B). Thus, the available observational evidence linked TLS presence to an approximately 46% lower adjusted hazard of death, although this association does not establish that TLSs are causal or therapeutically predictive.

Secondary outcomes were retained as distinct endpoints in the extraction and endpoint-specific displays (Figure 1C). Because each endpoint alone contributed too few compatible cohorts for a stable pooled estimate, an additional post hoc exploratory synthesis combined related PFS, DFS, and RFS endpoints only to assess direction (HR 0.648, 95% CI 0.411–1.021; prediction interval 0.393–1.070). Three cohorts reporting cause-specific or disease-specific survival yielded HR 0.463 (95% CI 0.264–0.813; prediction interval 0.239–0.897). The cross-endpoint synthesis was not interpreted as a single interchangeable clinical endpoint or as a substitute for the primary overall-survival analysis.

Risk-of-bias sensitivity analysis retained the direction and approximate magnitude of the overall-survival association after excluding studies with any high-risk QUIPS domain (three cohorts, 850 patients; HR 0.577, 95% CI 0.304–1.096), but the confidence and prediction intervals crossed the null. The pooled point estimate changed by 6.1%, indicating directional stability with the expected loss of precision after reducing the evidence base to three studies. Domain-level QUIPS judgments are shown in Figure S1.

### 3.2. TLS Prevalence Varies Across Definitions and Treatment Settings

Seven upfront, treatment-naive cohorts comprising 1137 patients and 371 TLS-positive cases contributed to the strict pathology prevalence model. The pooled prevalence was 37.9% (95% CI 21.9–57.1%), but heterogeneity was extreme (I^2^ = 96.5%) and the prediction interval ranged from 6.7% to 83.9% (Figure 1D). This interval, rather than the pooled mean alone, indicates that TLS detection depends strongly on anatomical location, sectioning strategy, marker panel, positivity threshold, and cohort context.

**Figure 2:**
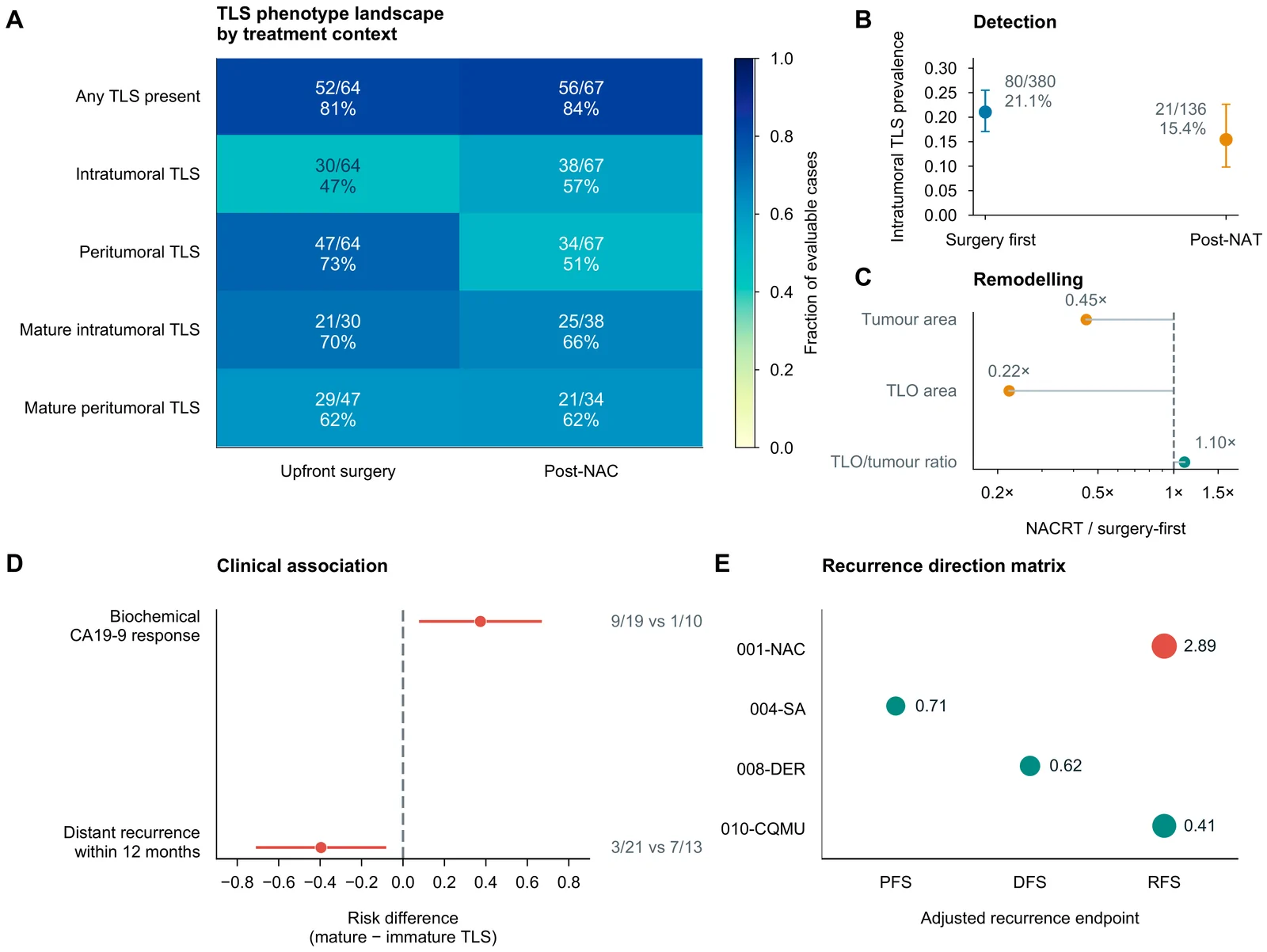
Treatment context and TLS maturation in PDAC. (**A**) TLS phenotype frequencies in upfront-surgery and post-neoadjuvant chemotherapy cohorts. Cell labels show the number of cases with the indicated phenotype over the relevant denominator and the corresponding percentage; the color scale represents this proportion. For the mature-TLS rows, denominators are cases with TLSs at the corresponding anatomical location. (**B**) Intratumoral TLS prevalence in surgery-first (blue) and post-neoadjuvant therapy (orange) cohorts. Points represent observed proportions, and vertical error bars show 95% confidence intervals. Labels indicate the numbers of TLS-positive cases and evaluated patients and the corresponding percentages. Estimates are displayed separately and should not be interpreted as a treatment effect because the underlying cohorts and TLS definitions were not exchangeable. (**C**) Descriptive ratios of tumour area, tertiary lymphoid organ (TLO) area, and the TLO-to-tumour area ratio in neoadjuvant chemoradiotherapy relative to surgery-first settings. The logarithmic horizontal axis expresses fold change. Orange points indicate ratios below 1, and the teal point indicates a ratio above 1. Horizontal segments connect each estimate to the dashed no-change reference at 1; these segments are not confidence intervals. (**D**) Associations between TLS maturation and biochemical CA19-9 response or distant recurrence within 12 months. Red points show risk differences, calculated as the proportion in the mature-TLS group minus that in the immature-TLS group; horizontal lines show 95% confidence intervals. Event counts are displayed in the same group order. The vertical dashed line denotes a risk difference of zero. (**E**) Study-specific adjusted hazard ratios for progression-free survival (PFS), disease-free survival (DFS), and recurrence-free survival (RFS). Values are shown alongside the circles; teal indicates HR < 1 and red indicates HR > 1. Blank cells indicate that no estimate is displayed for that cohort–endpoint combination. These panels provide descriptive treatment-context and clinical-association summaries, not estimates of a causal treatment effect. TLS, tertiary lymphoid structure; PDAC, pancreatic ductal adenocarcinoma; NAC, neoadjuvant chemotherapy; NAT, neoadjuvant therapy; NACRT, neoadjuvant chemoradiotherapy; HR, hazard ratio; CA19-9, carbohydrate antigen 19-9.

The two available post-neoadjuvant cohorts further demonstrated why treatment contexts could not be pooled mechanically. Observed TLS prevalence was 83.6% (56/67) in one any-location analysis and 15.4% (21/136) in an intratumoral analysis (Figure 2A,B). We therefore displayed study-specific estimates without a principal pooled value. The difference cannot be interpreted as a treatment effect because the TLS constructs and cohorts were not exchangeable.

**Figure 3:**
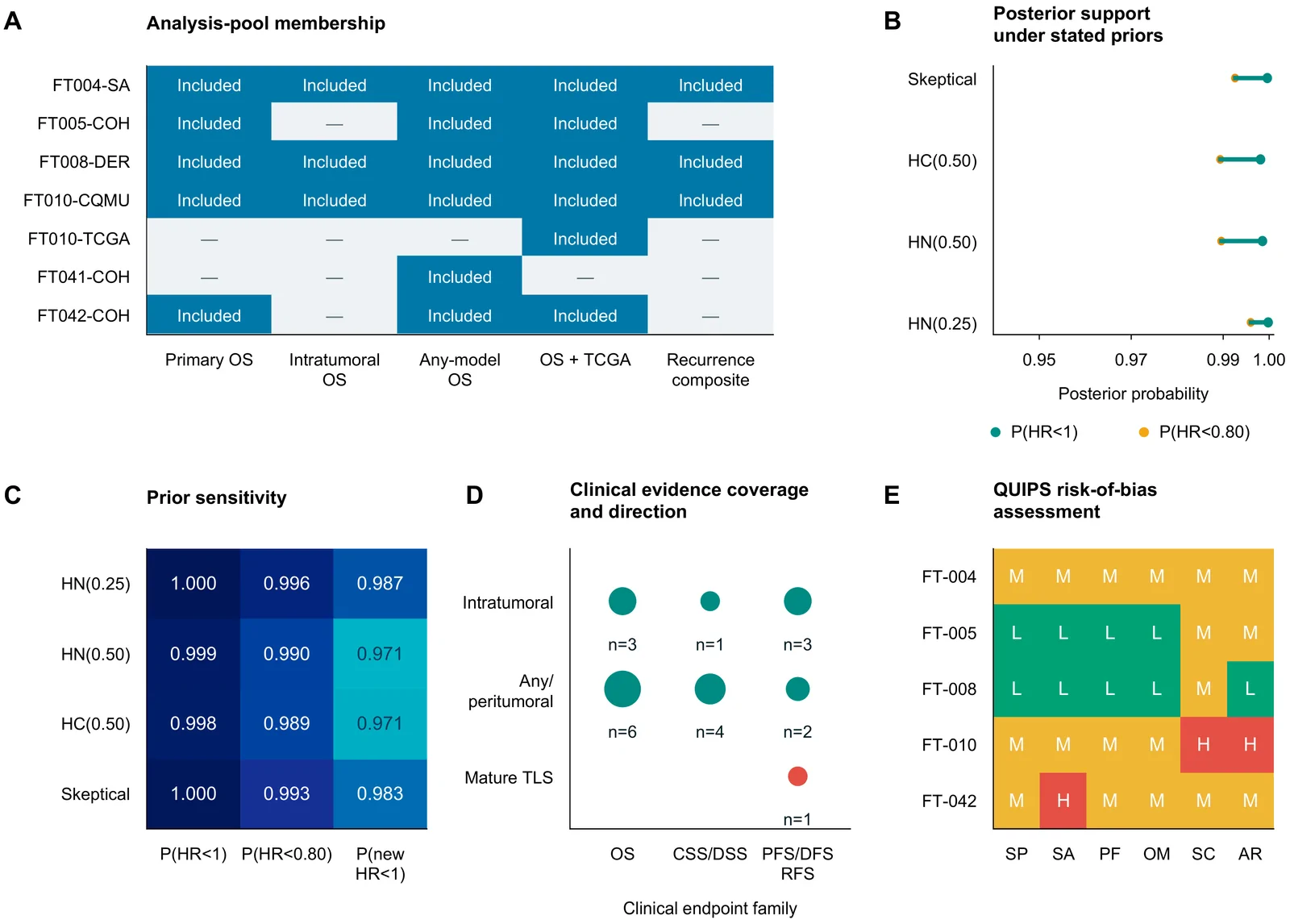
Robustness and evidence boundaries of the TLS–survival association. (**A**) Membership of the displayed clinical synthesis pools. Blue cells marked “Included” indicate inclusion, whereas pale cells marked with a dash indicate non-inclusion. (**B**) Posterior support under the stated prior distributions. Teal points represent P(HR < 1), and amber points represent P(HR < 0.80), where HR refers to the pooled mean association. Horizontal segments connect these two probabilities for each prior choice; they are not confidence or credible intervals. HN and HC denote half-normal and half-Cauchy priors for the between-study standard deviation, with the scale shown in parentheses. The HN(0.25), HN(0.50), and HC(0.50) models use a Normal(0, 2^2^) prior for the pooled mean log-HR. The skeptical model uses a Normal(0, 0.5^2^) effect prior and an HN(0.25) heterogeneity prior. (**C**) Corresponding prior-sensitivity probabilities. P(new HR < 1) is the posterior predictive probability of a favorable true association in a new study under the stated model. Cell values are rounded to three decimal places; darker blue shading indicates higher probabilities. These probabilities are model-dependent and are not frequentist *p* values. (**D**) Descriptive evidence coverage and direction by TLS construct and clinical endpoint family. Labels indicate the number of independent cohorts represented in each displayed construct–endpoint category. Teal indicates associations with HR < 1, and red indicates an association with HR > 1. Endpoint grouping is used for evidence display and does not imply that the endpoints are interchangeable. (**E**) Domain-level QUIPS risk-of-bias assessments for the five primary overall-survival cohorts. Green/L, yellow/M, and red/H denote low, moderate, and high risk of bias, respectively. SP, study participation; SA, study attrition; PF, prognostic factor measurement; OM, outcome measurement; SC, study confounding; AR, statistical analysis and reporting. QUIPS assesses risk of bias, not certainty of evidence. TLS, tertiary lymphoid structure; HR, hazard ratio; OS, overall survival; CSS, cause-specific survival; DSS, disease-specific survival; PFS, progression-free survival; DFS, disease-free survival; RFS, recurrence-free survival; TCGA, The Cancer Genome Atlas; QUIPS, Quality in Prognosis Studies.

An exploratory analysis restricted to explicitly intratumoral TLS retained a favorable overall-survival association (three cohorts, 753 patients; HR 0.643, 95% CI 0.422–0.979), although its prediction interval included the null (0.401–1.032; Figure 3A). Leave-one-study-out models for the primary overall-survival pool produced HRs from 0.488 to 0.616, indicating that no single study determined the favorable direction (Figure 1E). Together, the clinical evidence supported TLSs as a favorable prognostic marker while identifying location, maturation, and treatment as major sources of heterogeneity that required spatially resolved analysis.

### 3.3. Neoadjuvant Treatment Is Associated with Tissue-Composition Remodeling

**Figure 4:**
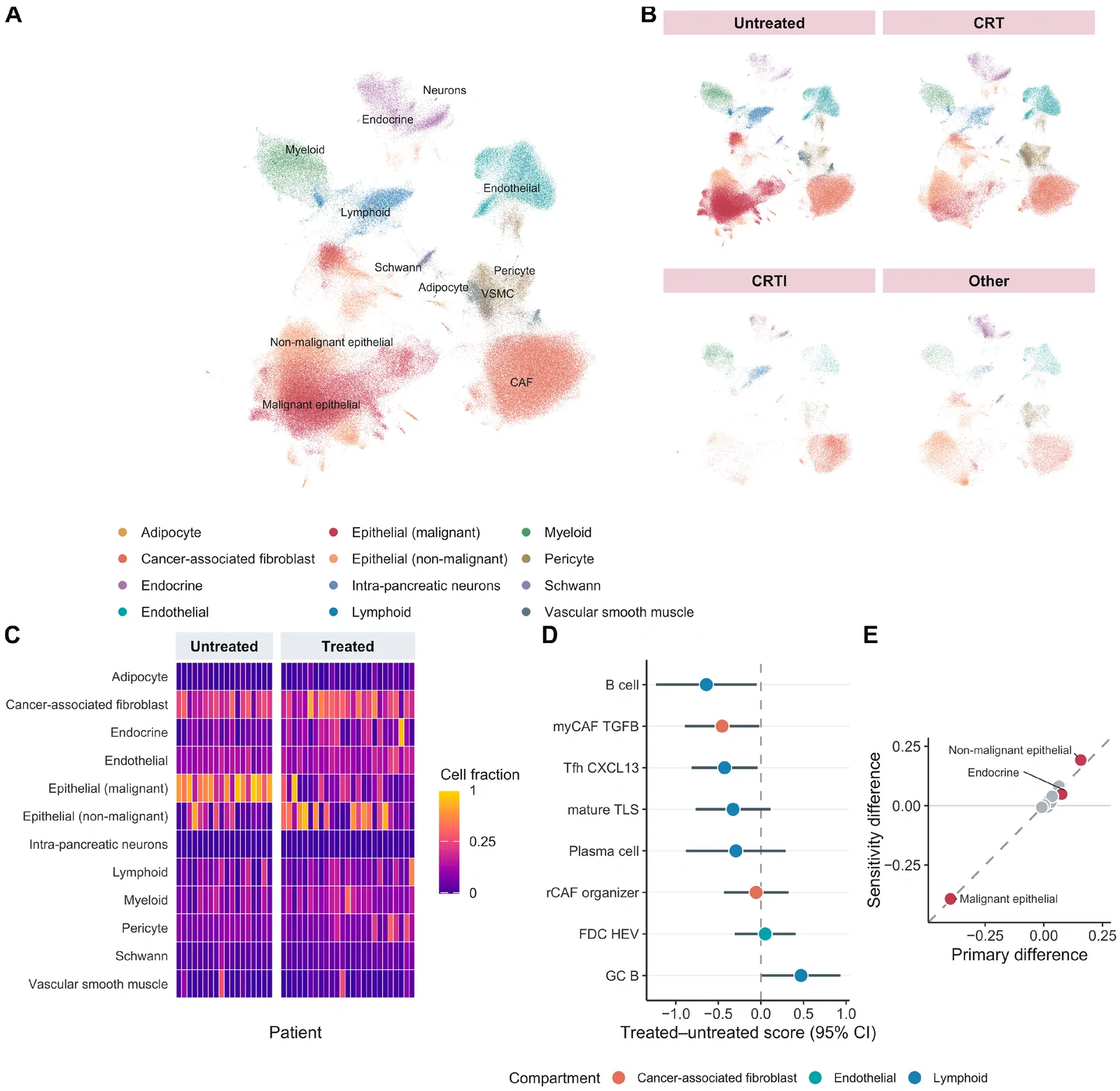
Treatment-associated tissue remodeling in GSE202051. (**A**) Complete cell-state map. (**B**) Treatment-stratified cell-state maps. (**C**) Specimen-level cell fractions across the 12 Level 1 populations. (**D**) Treated-minus-untreated score coefficients and 95% confidence intervals for the eight compartment-aware TLS programs, calculated using the supplied pseudobulk logCPM input. Points represent coefficients, and horizontal bars indicate 95% confidence intervals. Benjamini–Hochberg correction is applied jointly across the eight tests. (**E**) Comparison of cell-composition differences from the primary analysis and the sensitivity analysis restricted to specimens with at least 2000 nuclei. Each point represents a cell population; the dashed diagonal indicates equality between the two estimates. Specimens are the units of statistical inference; individual cells or nuclei are not treated as independent biological replicates.

Across the full GSE202051 object, Figure 4A shows the complete cell-state map, Figure 4B shows treatment-stratified composition, and Figure 4C summarizes patient-level composition across the principal populations. The malignant-cell fraction was the only composition result that remained significant after multiplicity correction: malignant cells represented a mean 49.46% of nuclei in untreated specimens and 9.66% in treated specimens (quasi-binomial BH-FDR = 0.00082).

All eight compartment-aware TLS programs were evaluated in Figure 4D using the retained pseudobulk logCPM input and joint BH correction. The five lymphoid programs had 10 untreated and 15 treated eligible specimens; the two CAF programs had 17 untreated and 24 treated specimens; and FDC/HEV had 18 untreated and 25 treated specimens. Zero-variance or non-finite gene scores were excluded before program averaging, as specified in the scoring procedure. No program passed BH correction in the treated-versus-untreated comparison (minimum BH-FDR = 0.116). Figure 4E compares the primary and specimen-threshold sensitivity estimates for cell composition; it is not an additional TLS-program test.

These data demonstrate treatment-associated remodeling of sampled tissue composition, dominated by a lower malignant-cell fraction, but do not show that treatment directly induces TLS programs. Sampling location, tumor content, regimen heterogeneity, and post-treatment selection may all contribute to the observed composition shift.

### 3.4. Pathology-Defined TLS Niches Reproducibly Enrich Mature Immune Programs

**Figure 5:**
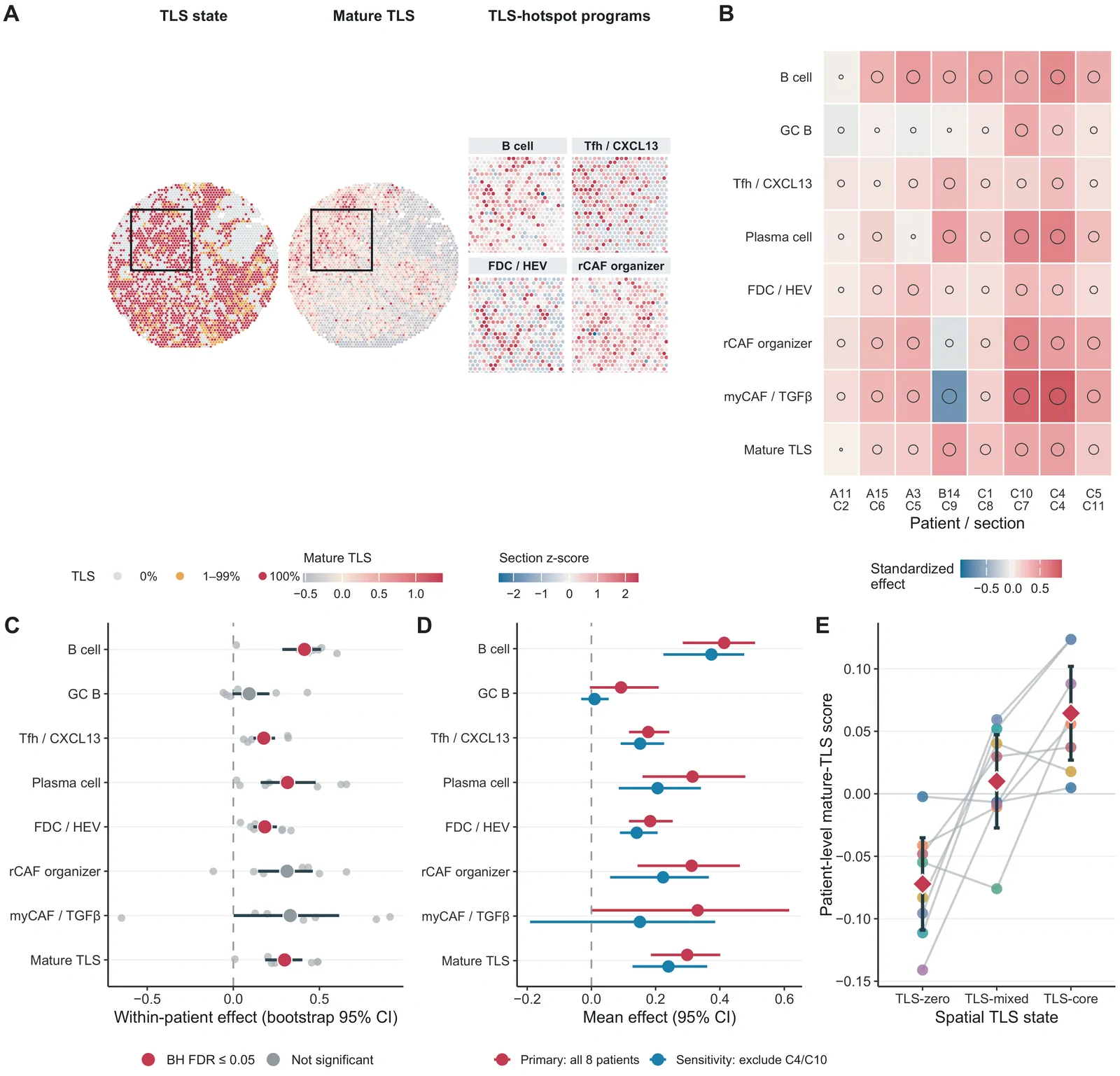
Pathology-anchored spatial enrichment of TLS programs in GSE277116. (**A**) Representative spatial maps of pathology-defined TLS status and mature-TLS scores. Boxed regions correspond to the enlarged maps of B-cell, Tfh/CXCL13, FDC/HEV, and rCAF-organizer programs, expressed as within-section z-scores. (**B**) Section-level standardized differences between TLS-positive and TLS-zero spots across eight programs. Red and blue indicate positive and negative differences, respectively; larger circles indicate greater absolute effects. Columns identify patients and their corresponding sections. (**C**) Patient-level standardized differences across eight tumor patients. Light-gray points represent individual patient estimates; larger points and horizontal bars indicate mean effects and bootstrap 95% confidence intervals. Red summary points indicate BH-FDR ≤ 0.05, whereas gray summary points indicate results that do not meet this threshold. (**D**) Comparison of primary estimates from all eight tumor patients (red) with sensitivity estimates after excluding patients C4 and C10 (blue; six patients). Points and horizontal bars indicate mean effects and bootstrap 95% confidence intervals. (**E**) Patient-level mean mature-TLS scores across TLS-zero, TLS-mixed, and TLS-core states. Colored points represent individual patients, and gray lines connect observations from the same patient. Red diamonds indicate means across patients; vertical bars indicate t-based 95% confidence intervals. Spatial maps and section-level effects are descriptive; patients, rather than sections or individual spots, are the independent units of statistical inference.

GSE277116 provided 12 Visium sections with matched pathology-derived TLS pixel annotations and a verified mapping to 10 patients. Two patients each contributed one tumor and one adjacent lymph-node section; we averaged section-level effects within patient before inference. Spatial maps showed that the fixed mature-TLS composite aligned with pathology-defined TLS-positive regions across heterogeneous sections (Figure 5A).

Section-level standardized differences were directionally consistent for B-cell, plasma-cell, rCAF organizer, Tfh/CXCL13, FDC/HEV, and mature-TLS programs, but these estimates remained descriptive because sections from the same patient were not independent (Figure 5B). At the patient level, the mature-TLS composite was higher in TLS-positive than TLS-zero spots across all 10 patients (mean standardized difference 0.201, bootstrap 95% CI 0.107–0.299; 9/10 positive; exact sign-flip BH-FDR = 0.00781; Figure 5C). The effect was also supported in the eight independent tumor patients (mean difference 0.236, 95% CI 0.118–0.368; 7/8 positive; BH-FDR = 0.0208). The four-patient lymph-node stratum was underpowered and did not pass correction.

Within tumor patients, B-cell, plasma-cell, rCAF organizer, Tfh/CXCL13, and FDC/HEV scores were all enriched in TLS-positive spots after BH correction. The GC B program was positive in six of eight patients but did not pass the within-family multiplicity threshold (BH-FDR = 0.0859). The myCAF/TGFβ program was also directionally positive in seven of eight tumor patients (BH-FDR = 0.0859), demonstrating that proximity-based TLS enrichment alone could not establish a simple organizer-versus-resistance fibroblast dichotomy.

### 3.5. Patient-Level TLS-Associated Programs Track Continuous TLS Burden

**Figure 6:**
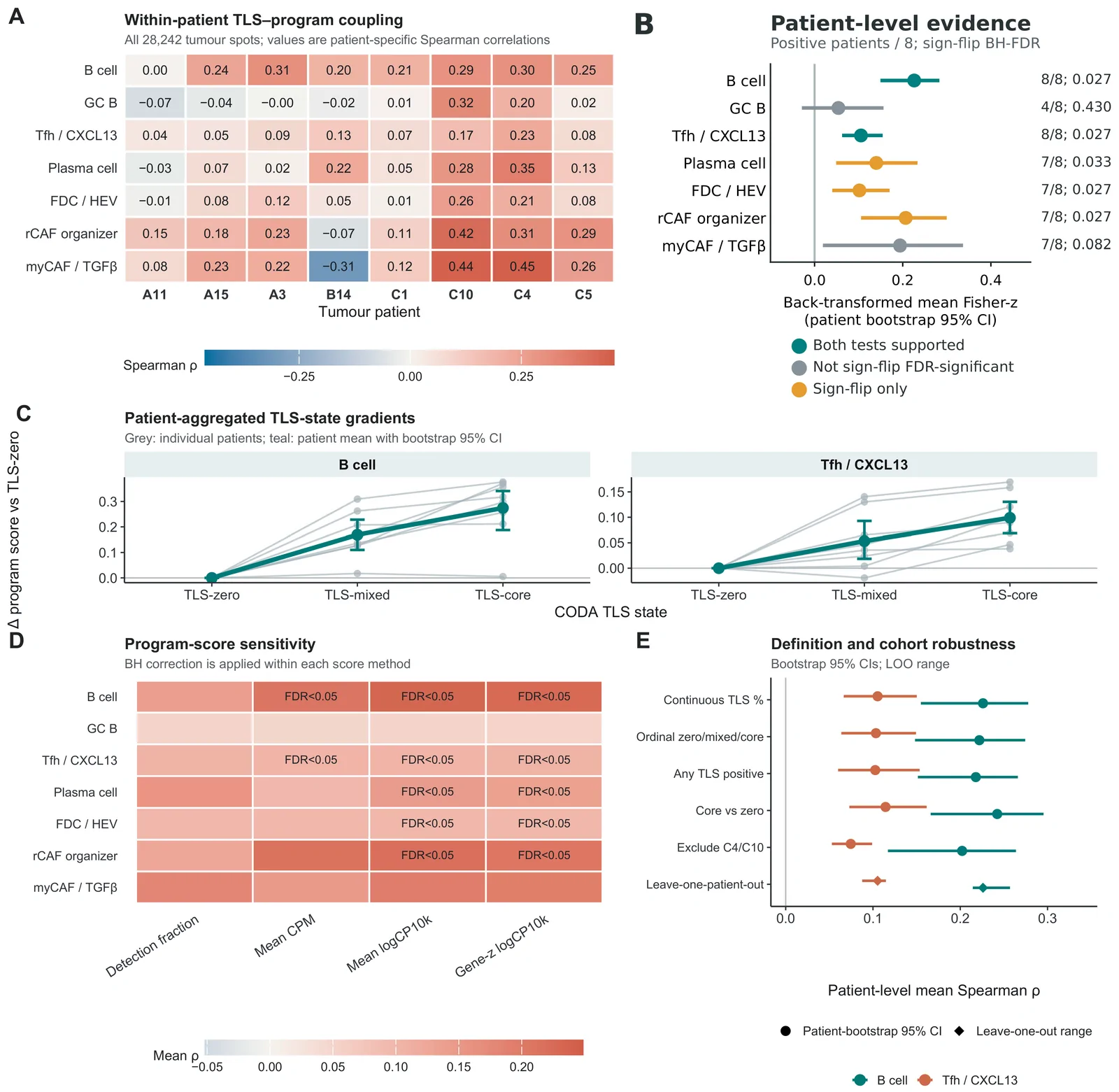
Patient-level TLS-associated programs in GSE277116. (**A**) Within-patient Spearman correlations between pathology-derived TLS percentage and seven fixed programs in eight tumor patients. (**B**) Patient-level correlations averaged on the Fisher-z scale and back-transformed to ρ, with pointwise 95% percentile confidence intervals obtained from 10,000 patient-level bootstrap resamples. Right-hand labels indicate the number of patients with positive correlations out of eight and BH-adjusted exact two-sided sign-flip *p* values across seven programs. Teal indicates significance in both the sign-flip and directional sign tests; amber indicates significance in the sign-flip test only; grey indicates no sign-flip significance after BH correction. For the rCAF-organizer program, ρ = 0.207, sign-flip BH-FDR = 0.0273, and directional sign-test BH-FDR = 0.0820. (**C**) Patient-aggregated changes in B-cell and Tfh/CXCL13 program scores across TLS-zero, TLS-mixed, and TLS-core states, relative to TLS-zero. Grey lines represent individual patients; teal points and error bars represent patient means and bootstrap 95% confidence intervals. (**D**) Sensitivity to four program-score constructions. Heatmap colors represent mean patient-level correlations; annotations identify BH-FDR < 0.05, with correction across seven programs within each scoring method. (**E**) Sensitivity to TLS definitions, exclusion of patients C4/C10, and leave-one-patient-out analysis. Circles and horizontal intervals show estimates and patient-bootstrap 95% confidence intervals; diamonds and horizontal ranges summarize leave-one-patient-out robustness. Patients are the independent statistical units. Confidence intervals are pointwise and are not multiplicity-adjusted. These analyses describe spatial associations and robustness, not cell–cell communication or causal recruitment.

To move beyond a binary spot contrast, we correlated pathology-derived TLS percentage with fixed cellular programs separately within each of the eight tumor patients and pooled Fisher-z-transformed correlations across patients. The resulting patient-by-program map revealed consistent positive relationships for B cells and Tfh/CXCL13, with more variable relationships for GC B, FDC/HEV, and fibroblast programs (Figure 6A).

The continuous TLS-burden results are summarized in Figure 6B. Using within-patient Spearman correlations, Fisher-z patient summaries, and exact two-sided patient sign-flip tests with BH correction across seven programs, B-cell and Tfh/CXCL13 programs were positive in all eight patients (mean rho = 0.226 and 0.105, respectively; sign-flip BH-FDR = 0.0273 for each) and also passed the directional sign-test sensitivity analysis. Plasma-cell, FDC/HEV, and rCAF-organizer programs showed positive magnitude-sensitive evidence (mean rho = 0.140, 0.102, and 0.207; sign-flip BH-FDR = 0.0328, 0.0273, and 0.0273, respectively) but did not pass the directional sign-test sensitivity threshold (BH-FDR = 0.0820 for each). The myCAF/TGFβ program was not significant after BH correction (mean rho = 0.194; sign-flip and directional BH-FDR = 0.0820). Figure 6C displays patient-aggregated TLS-zero, TLS-mixed, and TLS-core state gradients; Figure 6D tests score-construction sensitivity; and Figure 6E reports definition, cohort, and leave-one-patient-out robustness. These panels summarize the reported patient-level associations, state gradients, sensitivity analyses, and robustness analyses.

### 3.6. RCTD Sensitivity Analyses Support Relative Robustness but Not Cellular Recruitment

To assess whether RCTD-inferred spatial patterns depended on the inclusion of treated specimens in the single-nucleus reference, we repeated deconvolution with an untreated-only GSE202051 reference. Across 152 finite patient-by-cell-type primary effects, the original and untreated-only reference effects were concordant (Pearson r = 0.850; Spearman rho = 0.810; 125/152 [82.2%] with the same direction; Figure S4A). The corresponding effects from the TLS-high sensitivity analysis were similarly concordant (r = 0.858; 123/151 [81.5%] directional agreement). This supports the stability of relative effect patterns, but does not validate absolute deconvolution fractions or add a confirmatory cell-type claim.

Using image-coordinate six-nearest neighborhoods in eight tumor sections from eight independent patients, no cell type passed patient-level BH correction. The minimum exact sign-flip BH-FDR was 0.1641, shared by Ductal (atypical) and Hormone-negative neuroendocrine; the minimum BH-adjusted section-permutation empirical *p* value was 0.9837 (Figure S4B; Table S5). Thus, this exploratory analysis did not provide evidence of cell-type enrichment around TLSs under the random-label null. It is distinct from the within-spot TLS-positive versus TLS-zero associations.

### 3.7. Independent Spatial Analysis Separates rCAF and myCAF Relationships

We therefore analyzed GSE322767, which contains eight patient-level Visium CytAssist objects with 98,601 total spots, continuous lymphoid probability, and six deposited CAF program scores. The dataset did not provide a reliable categorical treatment assignment or a validated TLS-permissive-versus-TLS-resistant patient label. We used a continuous analysis in which within-patient Spearman correlations were the effect estimates and patients were the independent units.

**Figure 7:**
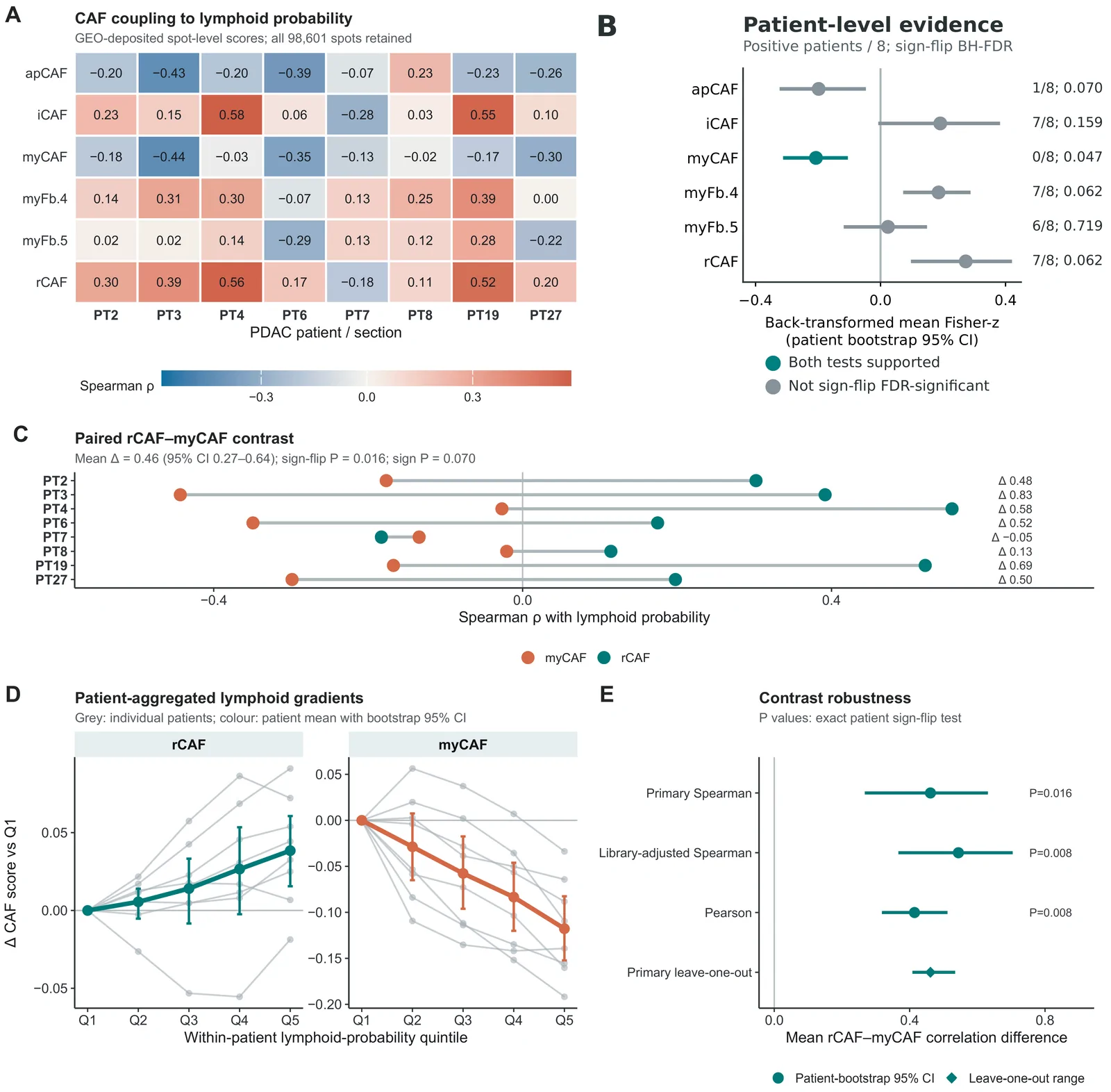
Patient-level CAF–lymphoid spatial associations in GSE322767. (**A**) Within-patient Spearman correlations between lymphoid probability and six deposited CAF program scores in eight patients. (**B**) Patient correlations averaged on the Fisher-z scale and back-transformed to ρ, with pointwise 95% percentile confidence intervals from 10,000 patient-level bootstrap resamples. Right-hand labels indicate the number of patients with positive correlations out of eight and BH-adjusted exact two-sided sign-flip *p* values across six programs. Teal indicates significance in both the sign-flip and directional sign tests; grey indicates no sign-flip significance after BH correction. (**C**) Paired within-patient rCAF and myCAF correlations. Connecting lines link estimates from the same patient; right-hand labels show the rCAF-minus-myCAF difference. (**D**) Patient-aggregated rCAF and myCAF score gradients across within-patient lymphoid-probability quintiles, expressed relative to the lowest quintile (Q1). Grey lines represent individual patients; colored points and error bars show patient means and bootstrap 95% confidence intervals. (**E**) Sensitivity of the rCAF-minus-myCAF correlation contrast to correlation method and library adjustment, together with leave-one-patient-out robustness. Circles and intervals show mean contrasts and bootstrap 95% confidence intervals; the diamond and horizontal range summarize leave-one-patient-out estimates. Reported *p* values are from exact two-sided patient-level sign-flip tests. Patients are the independent statistical units. Confidence intervals are not multiplicity-adjusted. These associations do not establish fibroblast lineage conversion or causal recruitment.

The patient-by-program map revealed opposing rCAF and myCAF gradients (Figure 7A). myCAF scores were negatively associated with lymphoid probability in all eight patients (back-transformed mean Fisher-z rho = −0.207, 95% CI −0.306 to −0.110; BH-FDR = 0.0469), whereas rCAF scores were positive in seven of eight patients (back-transformed mean Fisher-z rho = 0.273, 95% CI 0.103–0.415; unadjusted exact *p* = 0.0313, BH-FDR = 0.0625; Figure 7B). The direct within-patient rCAF-minus-myCAF correlation contrast was positive in seven patients, with a mean difference of 0.461 (median 0.510; exact sign-flip *p* = 0.0156; Figure 7C).

Leave-one-patient-out analysis retained a positive mean rCAF-minus-myCAF contrast regardless of the omitted patient (Figure 7E). A threshold-based top-versus-bottom lymphoid-probability sensitivity analysis supported lower myCAF scores in lymphoid-rich regions as a related sensitivity assessment (Figure 7D). Thus, an independent spatial cohort resolved a relative organizer-versus-myofibroblast axis that was obscured in the pathology-proximity analysis. Because these are spatial associations among deposited scores, they do not establish fibroblast lineage conversion or causal lymphocyte recruitment.

### 3.8. Hypothesis-Guided Chemokine Axes Co-Enrich in TLS-Positive Tumor Niches

**Figure 8:**
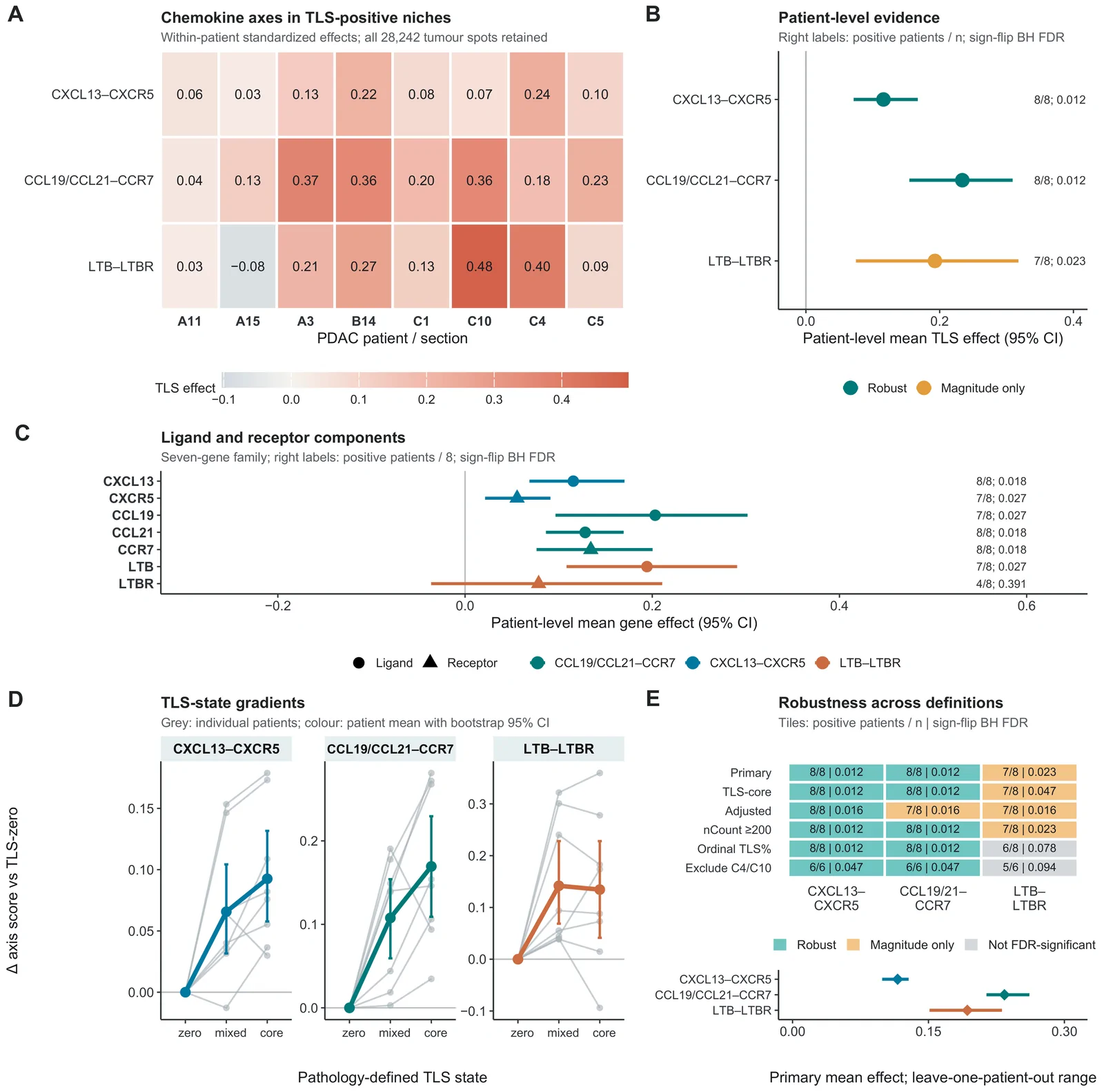
Patient-level chemokine-axis co-enrichment in pathology-defined TLS-positive tumor niches in GSE277116. (**A**) Within-patient standardized differences between TLS-positive and TLS-zero spots for three fixed axis scores in eight tumor patients. Heatmap values show patient-specific effects. (**B**) Mean patient-level axis effects with bootstrap 95% confidence intervals. Right-hand labels indicate positive patients/8 and BH-adjusted exact two-sided sign-flip *p* values across three axes. Teal indicates significance in both the sign-flip and directional sign tests; amber indicates significance in the sign-flip test only. (**C**) Mean patient-level effects for seven component genes, with bootstrap 95% confidence intervals. Circles denote ligands and triangles denote receptors; colors distinguish the three axes. Right-hand labels report positive patients/8 and sign-flip BH-FDR across seven genes. (**D**) Patient-aggregated axis-score gradients across TLS-zero, TLS-mixed, and TLS-core states, labeled zero, mixed, and core, respectively, relative to TLS-zero. Grey lines represent individual patients; colored points and error bars show patient means and bootstrap 95% confidence intervals. (**E**) Sensitivity to TLS definition, library adjustment, library-count filtering, ordinal TLS percentage, and exclusion of patients C4/C10. Tiles report positive patients/n and sign-flip BH-FDR, corrected across three axes within each analysis. Teal indicates significance in both tests; amber indicates sign-flip significance only; grey indicates no sign-flip significance after BH correction. The lower plot shows primary mean effects and leave-one-patient-out ranges. Patients are the independent statistical units. Confidence intervals are pointwise and not multiplicity-adjusted. Co-enrichment does not establish ligand–receptor binding, signaling direction, or sender/receiver identity.

We next tested three hypothesis-guided axes implicated in lymphoid organization: CXCL13–CXCR5, CCL19/CCL21–CCR7, and LTB–LTBR. All seven component genes were measured in every GSE277116 section. Patient-level effects in the eight tumor patients were predominantly positive across all three axes (Figure 8A).

The pooled standardized differences were 0.116 for CXCL13–CXCR5 (bootstrap 95% CI 0.071–0.166; BH-FDR = 0.0117), 0.236 for CCL19/CCL21–CCR7 (95% CI 0.156–0.314; BH-FDR = 0.0117), and 0.196 for LTB–LTBR (95% CI 0.073–0.334; BH-FDR = 0.0234; Figure 8B). At the component level, CXCL13, CXCR5, CCL19, CCL21, CCR7, and LTB passed correction, whereas LTBR did not (BH-FDR = 0.391; Figure 8C). The LTB–LTBR result was therefore primarily ligand driven.

Primary estimates and the contrast from the TLS-high sensitivity analysis were highly concordant across all patient-axis pairs (Pearson r = 0.995; Figure 8D). These results support spatial co-enrichment of lymphoid-organizing chemokine programs, most completely for CXCL13–CXCR5 and CCL19/CCL21–CCR7. They do not demonstrate ligand–receptor binding, directionality, receptor activation, or communication between specified cell types.

### 3.9. Exploratory Distance Gradients Refine Local Chemokine-Axis Enrichment

To test whether these spatial signals were concentrated toward the geometric center of pathology-defined TLSs, we performed a post hoc distance-gradient analysis in the same eight tumor patients. For each section, a TLS core was defined from spots with TLS percentage at least 50, and spots were grouped into core, near, mid, and far distance shells. Patient-level core-minus-far standardized differences were assessed using exact sign-flip testing across the three fixed axes (Figure S5).

LTB–LTBR was higher in TLS cores in all eight patients (mean standardized core-minus-far difference 0.224; BH-FDR = 0.023), and CCL19/CCL21–CCR7 was higher in seven of eight patients (0.129; BH-FDR = 0.047). CXCL13–CXCR5 was positive in six of eight patients (0.135) but did not pass correction (BH-FDR = 0.125). These findings refine the earlier TLS-positive-versus-TLS-negative comparison by showing localized co-enrichment for two axes. Because the distance shells, analysis, and visualizations were added as secondary extensions of the original harmonized reanalysis, they are reported as exploratory and do not alter the interpretation that expression co-enrichment cannot establish ligand–receptor binding, signaling direction, or causality.

### 3.10. Cross-Dataset Synthesis Defines a Supported Core and Explicit Uncertainty

**Figure 9:**
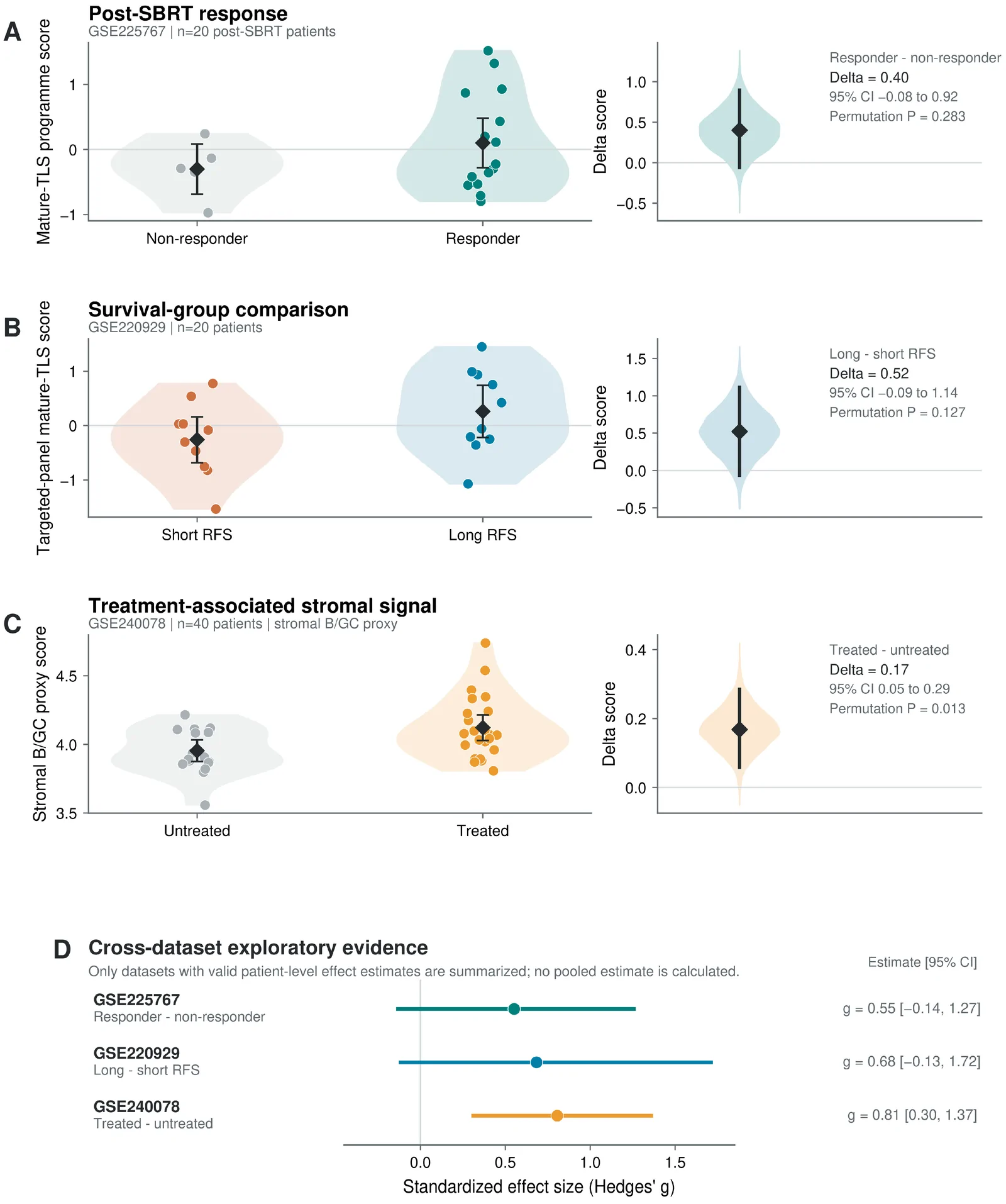
Exploratory cross-dataset assessments presented separately by cohort. (**A**) Post-SBRT response association in GSE225767 (20 patients), showing patient-level mature-TLS scores and the responder-minus-non-responder mean difference. (**B**) Long-versus-short recurrence-free groups in GSE220929 (20 patients), showing patient-level targeted-panel mature-TLS scores and the long-minus-short mean difference. (**C**) Treated-versus-untreated comparison in GSE240078 (40 patients), showing an exploratory two-gene stromal B/GC proxy and the treated-minus-untreated mean difference. In A–C, individual points represent patients; the right-hand distributions show bootstrapped mean differences, with 95% percentile confidence intervals from 7000 patient-level bootstrap resamples. Cohort-specific permutation *p* values are reported. (**D**) Standardized effects (Hedges’ g) and 95% percentile confidence intervals from 7000 patient-level bootstrap resamples for the same three contrasts. Points, whiskers, and numerical labels correspond to the retained standardized-effect results. The cohorts differ in endpoint, platform, score definition, and effect scale; the estimates are displayed for visual comparison only and are not pooled. GSE226840 is excluded from inferential panels because its 14 TLS microdissections originated from eight patients and a recoverable region-to-patient mapping was unavailable. These exploratory assessments do not establish independent validation or causal relationships. SBRT, stereotactic body radiotherapy; RFS, recurrence-free survival; B/GC, B-cell/germinal-center.

The exploratory cross-dataset assessments are shown separately by cohort in Figure 9. GSE225767 assessed post-SBRT response (*n* = 20 patients; *p* = 0.283), GSE220929 assessed long-versus-short recurrence-free groups (*n* = 20 patients; *p* = 0.127), and GSE240078 assessed a treated-versus-untreated stromal B/GC proxy (*n* = 40 patients; *p* = 0.013, explicitly exploratory). These three patient-level datasets differed in endpoint, score definition, platform, and effect scale and were not pooled. Figure 9D places their standardized effect estimates (Hedges’ g with 95% bootstrap confidence intervals) on a common display scale for visual comparison only; no pooled effect is estimated. GSE226840 was excluded from inferential cross-dataset testing because its 14 TLS microdissections arose from tumors from eight patients and a recoverable region-to-patient mapping was unavailable.

The targeted outcome assessment in GSE220929 was statistically unsupported. Long-term recurrence-free patients (*n* = 10) had a slightly higher fixed mature-TLS panel score than short-term recurrent patients (*n* = 10), but the effect was imprecise (Hedges’ g = 0.684; exact two-sided permutation *p* = 0.127). GSE225767 likewise did not show a statistically supported post-SBRT response association (exact *p* = 0.283). In contrast, the GSE240078 two-gene B/GC proxy differed between treated and untreated patients (permutation *p* = 0.013), but this post hoc proxy does not validate the full mature-TLS program. These heterogeneous results are therefore used to define the limits of cross-dataset transportability rather than to claim independent validation.

Across the external evidence landscape, the three patient-level datasets yielded heterogeneous findings, two of which were not statistically significant. They are presented as separate exploratory assessment contexts rather than as a unified validation set or mechanistic model. GSE226840 contributes no inferential effect estimate because patient-level dependence could not be reconstructed. No panel in Figure 9 establishes causal ordering among TLSs, stromal states, treatment, and clinical outcome. The public molecular datasets and their analytic roles are summarized in Table 2.

## 4. Discussion

This study integrates clinical pathology, single-nucleus composition, and patient-aware spatial transcriptomics to define what can and cannot currently be concluded about TLSs in PDAC. Four findings converge. First, pathology-defined TLSs are associated with longer overall survival across independent resection cohorts. Second, pathology-positive spatial niches showed co-enrichment of B-cell, Tfh/CXCL13, plasma-cell, FDC/HEV, and reticular-fibroblast programs, with varying consistency across estimands and sensitivity analyses. Third, an independent spatial cohort showed stronger alignment of the rCAF program than the myCAF/TGFβ program with continuous lymphoid probability; this remains an associative comparison. Fourth, chemokine axes associated with lymphoid recruitment co-enriched in TLS-positive regions, but component-level results and heterogeneous cross-dataset evidence delimit the mechanistic claim.

The clinical synthesis strengthens prior observations that lymphoid organization identifies a favorable PDAC subset. The adjusted overall-survival association was consistent across five cohorts, remained directionally stable in leave-one-out analyses, and changed little after excluding studies with high-risk QUIPS domains. Yet the evidence remains observational. TLSs may be a marker of a more immunogenic tumor, favorable tumor biology, treatment responsiveness, or host immune competence rather than the direct cause of longer survival. Residual confounding by stage, treatment, sampling, and pathology assessment cannot be removed through meta-analysis.

The extraordinary prevalence heterogeneity is biologically informative. Studies sampling intratumoral versus peritumoral tissue, using one section versus extensive sampling, or requiring germinal centers versus any lymphoid aggregate are not measuring the same phenotype. The 6.7–83.9% prediction interval in upfront cohorts and the discordant post-neoadjuvant estimates argue against a universal PDAC TLS prevalence. They instead support a model in which TLSs emerge along continuous axes of anatomical location, maturation, treatment context, and stromal permissiveness.

Patient-aware spatial analysis substantially changes the strength of inference. Thousands of spots can map a niche with high resolution, but they do not create thousands of independent biological replicates. By first estimating effects within each patient and then using exact patient-level sign-flip tests, we identified reproducible TLS enrichment without pseudoreplication. This approach confirmed the mature-TLS composite in both the ten-patient overall set and the eight-patient tumor subset. It also distinguished consistent B-cell and Tfh associations from less stable GC B and FDC burden relationships.

The RCTD analyses provide an additional boundary: reference composition did not materially alter relative TLS effects, but the neighborhood permutation analysis found no corrected evidence of immediate cellular enrichment. Accordingly, RCTD is used here to assess robustness and spatial association, not migration, recruitment, or absolute cell fractions.

The combined CAF analyses suggest that fibroblast state may gate lymphoid organization. In GSE277116, rCAF and myCAF programs could both be elevated near pathology-defined TLSs, possibly reflecting mixed stromal states after immunotherapy or spatial averaging within Visium spots. GSE322767 offered a more discriminating continuous lymphoid gradient: rCAF was preferentially aligned and myCAF was consistently opposed. This agrees with experimental evidence that myofibroblast differentiation can restrict the reticular fibroblast program needed for lymphoid organization [23]. Nevertheless, our reanalysis does not test lineage conversion, TGFβ dependence, or LTBR responsiveness. The direct rCAF-minus-myCAF contrast is a robust spatial association, not a causal perturbation.

The chemokine results similarly require disciplined interpretation. CXCL13–CXCR5 and CCL19/CCL21–CCR7 had support on both ligand and receptor sides, consistent with B-cell and lymphocyte positioning. LTB–LTBR was less complete because LTBR itself was unsupported. Co-expression of a ligand and receptor in the same pathology-defined niche does not identify sender and receiver cells, prove physical interaction, or establish downstream signaling. Higher-resolution spatial single-cell assays, protein localization, and perturbation will be required to convert these axes from correlative biomarkers into therapeutic mechanisms.

The treatment analysis provides context rather than proof of TLS induction. The marked reduction in malignant-cell fraction among treated GSE202051 specimens is compatible with extensive tissue remodeling, but no eligible TLS module passed multiplicity correction. The analysis cannot distinguish drug effects from sampling location, response selection, or regimen heterogeneity. Likewise, the two post-neoadjuvant prevalence reports used incompatible TLS definitions. These findings support treatment as an important stratification variable, while cautioning against labeling TLSs as treatment induced without paired specimens or randomized comparisons.

The cross-dataset targeted-panel results are important because they limit generalizability. A mature-TLS score did not significantly separate long-term recurrence-free and short-term recurrent patients in GSE220929, and the post-SBRT response association in GSE225767 was also statistically unsupported. GSE240078 provided an exploratory positive result for a two-gene B/GC proxy, not the full mature-TLS program. GSE226840 was not used for inferential maturation testing because 14 TLS regions were nested within eight patients and region-to-patient membership could not be recovered. Together, these constraints challenge the notion that one reduced signature can serve as a universal TLS biomarker. Morphology, spatial organization, cellular composition, and treatment context may be inseparable components of TLS biology.

For translation, the data support pathology-anchored TLS assessment as a prognostic-enrichment strategy, not yet as a treatment-selection biomarker. A prospective study should combine standardized whole-slide TLS detection, maturation scoring, spatial rCAF/myCAF phenotyping, and prespecified clinical endpoints. Serial or paired tissue would be required to establish treatment induction. Mechanistic trials should test whether suppressing myofibroblast programming while activating lymphoid-organizer pathways increases mature TLSs and antitumor immunity. Critically, those trials should treat TLS formation, functional immune activation, and clinical benefit as separate endpoints.

In conclusion, PDAC TLSs identify a clinically favorable and spatially organized immune state. The supported core comprises pathology-associated survival; patient-level mature-TLS enrichment; reproducible B-cell and Tfh/CXCL13 associations; less directionally consistent plasma-cell, FDC/HEV, and rCAF signals; and chemokine-program co-enrichment. Treatment induction, direct ligand–receptor communication, causal fibroblast control, and stand-alone clinical prediction remain open questions. Preserving these boundaries turns a heterogeneous body of literature into a testable framework for prospective PDAC immunobiology.

### Limitations

This work is a secondary analysis of observational studies and public molecular datasets. Most clinical cohorts were retrospective and were therefore vulnerable to selection bias, missing or inconsistently measured covariates, nonuniform treatment and follow-up, and incomplete control of confounding. Moreover, TLS presence, density, area, anatomical location, maturation, and marker-defined lymphoid aggregates are biologically related but non-interchangeable prognostic constructs. The clinical meta-analysis included few cohorts per endpoint and remains susceptible to residual confounding, selective reporting, and pathology measurement bias. Formal small-study-effect tests were not performed because each synthesis contained fewer than ten studies. Two reviewers independently completed screening, full-text assessment, data extraction, and QUIPS assessment; disagreements and the final analysis-pool lock were resolved by consensus with third-reviewer adjudication when required.

Public molecular datasets were generated for different primary objectives and used different platforms, treatments, tissue contexts, and annotations. GSE277116 contained only eight independent tumor patients with pathology annotations; the lymph-node stratum contained four patients. GSE322767 contained eight patients and deposited program scores but lacked reliable treatment labels. GSE202051 was cross-sectional, and treated versus untreated specimens were not paired. The targeted panels incompletely measured the full program. In GSE226840, 14 TLS microdissections arose from eight patients, but the region-to-patient mapping was unavailable, precluding donor-aware inference. These limitations reduce power, constrain transportability, and prevent the external datasets from being interpreted as independent validation cohorts.

Spatial correlations and co-enrichment cannot establish cell lineage, ligand–receptor binding, signaling direction, or causality. Visium spots may contain multiple cell types, and correlations among bounded or deconvolution-derived programs may partly reflect compositional structure. Likewise, the GSE202051 cell fractions sum to one, so an apparent change in one population may partly reflect reciprocal changes in other populations; separate quasi-binomial models and the ≥2000-cell sensitivity analysis do not remove this constraint. The unpaired treated-versus-untreated comparison therefore cannot establish treatment-induced lineage change. No new experimental validation was performed. These limitations motivate prospective pathology standardization, spatial single-cell validation, and functional perturbation rather than broader claims from the present data.

RCTD fractions are model-based estimates and depend on the selected reference. Although the untreated-only reference sensitivity analysis was directionally concordant with the primary analysis, it cannot validate absolute fractions. The 999-fold within-section neighborhood permutation analysis has limited power with eight patients and its negative result rules out neither subtler nor nonlocal cellular organization.

The clinical evidence base included studies of TLS-related immune phenotypes and outcome in PDAC [38,43,46]. Additional cohorts assessed spatial leukocyte features, tumor-microenvironment immune factors, and post-neoadjuvant TLS maturity [37,44,45]. Reports of stromal immune localization, vaccine-platform treatment, and long-term-survivor B-cell spatial organization provided complementary clinical and translational context [42,50,52].

## 5. Conclusions

Pathology-defined TLSs are associated with favorable overall survival in PDAC and identify spatially organized immune niches. Patient-aware analyses support mature-TLS enrichment and reproducible B-cell and Tfh/CXCL13 associations, with less directionally robust plasma-cell, FDC/HEV, and rCAF signals, together with an rCAF-over-myCAF spatial comparison and chemokine-program co-enrichment. These associations do not establish direct signaling, cellular recruitment, treatment induction, or clinical predictiveness; prospective, spatially resolved validation is required.

## Figures and Tables

**Table 1:** Clinical evidence base.

Study	Country	Design	Recruitment Period	Population/Treatment	Cohort Size (s)	TLS Assessment	Eligible Outcomes	Follow-Up	Synthesis Role
Nabeya et al., 2026 [37]	Japan	Retrospective surgical cohort; Retrospective post-NAC surgical cohort	January 2010 to March 2024; 2016 to March 2024	Upfront/treatment-naïve resection; Post-NAC/NAT resection	Upfront surgery (*n* = 64); Neoadjuvant chemotherapy followed by surgery (*n* = 67)	Overall TLS/iTLS/periTLS	RFS	NR	Quantitative evidence
Hu et al., 2025 [38]	China; United States/multicentre	Retrospective surgical cohort; Public dataset validation cohort	January 2017 to August 2023	Upfront/treatment-naive resection	CQMU pathology cohort (*n* = 65); TCGA-PAAD pathology-classified cohort (*n* = 143)	Intratumoral TLS presence vs. absence	OS; RFS	NR	Quantitative evidence
Lehmann et al., 2025 [20]	Germany	Retrospective multicentre surgical cohort	2006 to 2018	Upfront/treatment-naive resection	Cologne/Munich resected treatment-naive cohort (*n* = 110)	TLS area score; high ≥ 66th percentile vs. low < 33rd percentile	OS	NR	Quantitative evidence
Wang et al., 2025 [39]	China	Retrospective surgical cohort	2020 to 2023	Upfront/treatment-naive resection	Dalian Medical University cohort (*n* = 69)	Germinal-center-positive TLSs	OS	NR	Quantitative evidence
Yan et al., 2025 [40]	China	Retrospective surgical imaging-pathology cohort	2014 to 2023	Upfront/treatment-naive resection	Resected PDAC cohort (*n* = 266)	TLS presence in plotted survival groups; Stage-stratified TLS presence	OS	NR	Quantitative evidence
Chen, 2024 [41]	China	Retrospective single-institution surgical cohort	NR	Upfront/treatment-naive resection	CAMS/PUMC resected PDAC cohort (*n* = 282)	TLS presence, intratumoral/peritumoral location and abundance	PFS; DSS	NR	Quantitative evidence
Heumann et al., 2023 [42]	United States	Platform clinical trial correlative cohort	March 2016 to September 2021	Immunotherapy-treated clinical cohort	GVAX + nivolumab + urelumab platform trial (*n* = 23)	CD8^+^CD137^+^ cell density within TLA	DFS; OS	NR	Quantitative evidence
Tanaka et al., 2023 [18]	Japan	Retrospective consecutive surgical cohort	April 2008 to March 2017	Upfront/treatment-naive resection	Kobe University resected PDAC cohort (*n* = 162)	Any TLS presence	CSS; OS	Median 26 months (range 1 to 122)	Quantitative evidence
Zou et al., 2023 [19]	China	Retrospective surgical cohort; Retrospective post-NAT surgical cohort; Spatial pathology subset	2013 to 2019	Upfront/treatment-naive resection; Post-NAC/NAT resection	Surgery-alone cohort (*n* = 380); Neoadjuvant-treated cohort (*n* = 136); NAT peri/intratumoral spatial subset (*n* = 22)	Intratumoral TLS presence; Peritumoral TLS presence; Peritumoral TLS maturity among peri-TLS-positive cases	OS; PFS	NR	Quantitative evidence
Ahmed et al., 2022 [43]	Germany; Public dataset	Retrospective surgical cohort; Public transcriptomic survival validation cohort	March 2007 to July 2011	Upfront/treatment-naive resection; Public transcriptomic validation dataset	Heidelberg resected PDAC cohort (*n* = 55); Xena public transcriptomic survival cohort (*n* = 183)	Peritumoral TLS presence	OS	NR	Quantitative evidence
Gunderson et al., 2021 [16]	United States	Retrospective surgical cohort	NR	Upfront/treatment-naive resection	Resectable primary-surgery cohort (*n* = 63)	Early TLS presence	OS; PFS	NR	Quantitative evidence
Kiryu et al., 2021 [44]	Japan	Retrospective surgical cohort	January 2005 to March 2019	Upfront/treatment-naive resection	R0-resected PDAC cohort (*n* = 85)	PD-1-positive vs. PD-1-negative tertiary lymphoid aggregates	OS; DFS	Minimum_reported 2 years	Quantitative evidence
Liudahl et al., 2021 [45]	United States	Retrospective spatial pathology cohort	NR	Upfront/treatment-naive resection	Untreated discovery pathology cohort (*n* = 104)	TLS presence; HEV status across treatment-naive TLS; Full-panel TLS image-cytometry subsets	OS	NR	Narrative evidence
Zheng et al., 2021 [17]	United States	Clinical trial correlative cohort	July 2008 to February 2015	Vaccine-treated clinical cohort	GVAX neoadjuvant/adjuvant vaccine cohort (*n* = 66)	Induced TLA presence/size and immune features	OS; DFS	NR	Quantitative evidence
Kuwabara et al., 2019 [46]	Japan	Retrospective surgical cohort; Retrospective post-NACRT surgical cohort	January 2009 to December 2015	Upfront/treatment-naive resection; Post-NAC/NAT resection	Surgery-first stratum (*n* = 93); Neoadjuvant chemoradiotherapy stratum (*n* = 47)	TLO/tumor area ratio	OS	Median 749.5 days	Quantitative evidence
Masugi et al., 2019 [47]	United States	Retrospective surgical cohort	1991 to 2015	Upfront/treatment-naive resection	Resected PDAC cohort (*n* = 214)	Extensive vs. minimal/absent peritumoral TLSs	CSS	Median 27 months (range 1.8 to 265.4)	Narrative evidence
Wartenberg et al., 2018 [48]	Switzerland	Retrospective surgical molecular-pathology cohort	2002 to 2011	Upfront/treatment-naive resection	Molecularly profiled resected PDAC cohort (*n* = 110)	TLT presence	OS	NR	Quantitative evidence
Stromnes et al., 2017 [49]	United States	Translational tissue cohort; Translational mixed-treatment tissue cohort	NR	Mixed or incompletely reported treatment context	Small resected PDA survival subset (*n* = 14); All PDA tissues with TLS classification (*n* = 20)	TLS presence in survival subset; TLS presence in all PDA tissues	OS	NR	Quantitative evidence
Castino et al., 2016 [15]	Italy	Retrospective consecutive surgical cohort	February 2010 to December 2012	Upfront/treatment-naive resection	Humanitas non-metastatic resected PDAC cohort (*n* = 104)	Median immune densities (IRA%)	DSS	Mean 563.4 days	Quantitative evidence
Diana et al., 2016 [50]	Germany	Retrospective whole-mount surgical cohort	2009 to 2014	Upfront/treatment-naive resection	Adjuvant-treated resected PDAC cohort (*n* = 145)	Lymphoid aggregate presence	OS; PFS; LPFS; DMFS	Mean 20 months (range 2 to 69)	Narrative evidence
Poschke et al., 2016 [51]	Germany	Retrospective surgical immune-pathology cohort	NR	Upfront/treatment-naive resection	Heidelberg resected PDAC immune cohort (*n* = 127)	TLS presence vs. absence	OS	NR	Narrative evidence
Hiraoka et al., 2015 [14]	Japan	Retrospective surgical cohort; Retrospective validation surgical cohort	1990 to 2004; 2005 to 2009	Upfront/treatment-naive resection	Derivation resected PDC cohort (*n* = 308); Independent validation cohort (*n* = 226)	TLO grade	OS; DFS	Median 17.6 months (range 2.6 to 201); Median 21.6 months (range 1.9 to 99.6)	Quantitative evidence

**Table 2:** Public molecular datasets and analytic roles.

Dataset	Modality	Analytic Material	Primary Unit	Role	Principal Result	Evidence Boundary	Main Figure
GSE226840	NanoString targeted expression of laser-microdissected regions	19 regions: 14 TLS microdissections (CD23-negative, CD23-low, CD23-high) and 5 adjacent-normal samples; TLS regions derived from tumors from 8 patients	Dataset-level audit only; donor-aware inferential unit not recoverable	Gene-coverage and TLS-category audit; excluded from inferential cross-dataset testing	No inferential effect estimated	Region-to-patient mapping unavailable; region-level *p* values would violate independence and were therefore not reported	Not included in inferential Figure 9 panels
GSE277116	10x Visium spatial transcriptomics with pathology TLS annotation	12 annotated sections; 10 patients overall and 8 tumor patients	Patient after within-patient section aggregation	Primary spatial TLS analysis, cellular ecology, and chemokine-axis analysis	Mature-TLS enrichment supported; B-cell/Tfh ecology supported; three hypothesis-guided chemokine axes BH-FDR < 0.05	Spatial co-enrichment is associative and does not establish direct cell–cell communication	Figure 5, Figure 6 and Figure 8
GSE202051	Single-nucleus RNA sequencing	43 PDAC specimens: 18 untreated and 25 treated	Specimen	Treatment-associated tissue-remodeling analysis	Malignant-cell fraction differed by treatment (BH-FDR = 0.00082); no eligible TLS module passed BH correction	Treatment contrast is sensitive to sampling and tumor content and is not a direct TLS contrast	Figure 4
GSE322767	10x Visium CytAssist FFPE spatial transcriptomics	Tumor sections from 8 patientss	Patient	CAF-TLS spatial-ecology analysis	rCAF-minus-myCAF correlation difference 0.461; 7/8 positive; exact *p* = 0.0156; leave-one-out direction stable	rCAF alone showed a positive association that did not pass multiplicity correction (BH-FDR = 0.0625)	Figure 7
GSE220929	NanoString PanCancer Immune Panel	20 resected PDAC patients: 10 long-term recurrence-free and 10 short-term recurrent	Patient	Exploratory cross-dataset outcome assessment	Mature-TLS score difference was not statistically supported (Hedges g = 0.684; exact two-sided permutation *p* = 0.127)	Extreme-group design, small sample, and targeted panel preclude unbiased time-to-event estimation	Figure 9
GSE225767	Targeted expression after stereotactic body radiotherapy	20 post-SBRT PDAC samples: 15 responders and 5 non-responders	Patient	Exploratory cross-dataset response assessment	Mature-TLS score difference not statistically supported (exact *p* = 0.283)	Unpaired observational response association; does not establish a treatment-induced effect	Figure 9
GSE240078	Spatially resolved stromal expression	40 patients: 23 treated and 17 untreated	Patient	Exploratory cross-dataset stromal assessment	Two-gene B/GC proxy differed by treatment (permutation *p* = 0.013)	Post hoc two-gene proxy; does not validate the full mature-TLS program	Figure 9

## Data Availability

The public datasets analyzed in this study are accessible under the accession identifiers cited in the manuscript. Analysis scripts, processed input and result tables, and session information are provided in Supplementary Code and Data. Some upstream source files must be obtained from their original public repositories. No identifiable participant data are included.

## Supplementary Materials

The following supporting information can be downloaded at: https://www.mdpi.com/article/10.3390/biomedicines14102287/s1, Supplementary Methods S1–S3; Supplementary Figures S1–S5; Supplementary Tables S1–S5; the PRISMA 2020 checklist; and Supplementary Code and Data, including analysis scripts, processed analytical outputs, input manifests, SHA-256 records, and session-information files.

## References

[1] Dieu-Nosjean M., Goc J., Giraldo N.A., Sautès-Fridman C., Fridman W.H. (2014). Tertiary lymphoid structures in cancer and beyond. Trends Immunol..

[2] Sautès-Fridman C., Petitprez F., Calderaro J., Fridman W.H. (2019). Tertiary lymphoid structures in the era of cancer immuno-therapy. Nat. Rev. Cancer.

[3] National Cancer Institute Cancer Stat Facts: Pancreatic Cancer. Surveillance, Epidemiology, and End Results Program; Data from SEER 21 (Excluding Illinois). https://seer.cancer.gov/statfacts/html/pancreas.html.

[4] Öhlund D., Handly-Santana A., Biffi G., Elyada E., Almeida A.S., Ponz-Sarvise M., Corbo V., Oni T.E., Hearn S.A., Lee E.J. (2017). Distinct populations of inflammatory fibroblasts and myofibroblasts in pancreatic cancer. Cancer. J. Exp. Med..

[5] Biffi G., Oni T.E., Spielman B., Hao Y., Elyada E., Park Y., Preall J., Tuveson D.A. (2019). IL1-Induced JAK/STAT Signaling Is Antagonized by TGFβ to Shape CAF Heterogeneity in Pancreatic Ductal Adenocarcinoma. Cancer Discov..

[6] Elyada E., Bolisetty M., Laise P., Flynn W.F., Courtois E.T., Burkhart R.A., Teinor J.A., Belleau P., Biffi G., Lucito M.S. (2019). Cross-Species Single-Cell Analysis of Pancreatic Ductal Adenocarcinoma Reveals Antigen-Presenting Cancer-Associated Fibroblasts. Cancer Discov..

[7] Cumming J., Maneshi P., Dongre M., Alsaed T., Dehghan-Nayeri M.J., Ling A., Pietras K., Patthey C., Öhlund D. (2025). Dis-secting FAP+ Cell Diversity in Pancreatic Cancer Uncovers an Interferon-Response Subtype of Cancer-Associated Fibroblasts with Tumor-Restraining Properties. Cancer Res..

[8] Özdemir B.C., Pentcheva-Hoang T., Carstens J.L., Zheng X., Wu C., Simpson T.R., Laklai H., Sugimoto H., Kahlert C., Novitskiy S.V. (2014). Depletion of Carcinoma-Associated Fibroblasts and Fibrosis Induces Immunosuppression and Accel-erates Pancreas Cancer with Reduced Survival. Cancer Cell.

[9] Feig C., Jones J.O., Kraman M., Wells R.J.B., Deonarine A., Chan D.S., Connell C.M., Roberts E.W., Zhao Q., Caballero O.L. (2013). Targeting CXCL12 from FAP-expressing carcinoma-associated fibroblasts synergizes with anti–PD-L1 immuno-therapy in pancreatic cancer. Proc. Natl. Acad. Sci. USA.

[10] Sherman M.H., Yu R.T., Engle D.D., Ding N., Atkins A.R., Tiriac H., Collisson E.A., Connor F., Van Dyke T., Kozlov S. (2014). Vitamin D Receptor-Mediated Stromal Reprogramming Suppresses Pancreatitis and Enhances Pancreatic Cancer Therapy. Cell.

[11] Bailey P., Chang D.K., Nones K., Johns A.L., Patch A., Gingras M., Miller D.K., Christ A.N., Bruxner T.J.C., Quinn M.C. (2016). Genomic analyses identify molecular subtypes of pancreatic cancer. Nature.

[12] Puleo F., Nicolle R., Blum Y., Cros J., Marisa L., Demetter P., Quertinmont E., Svrcek M., Elarouci N., Iovanna J.L. (2018). Stratification of Pancreatic Ductal Adenocarcinomas Based on Tumor and Microenvironment Features. Gastroenterology.

[13] Peng J., Sun B., Chen C., Zhou J., Chen Y., Chen H., Liu L., Huang D., Jiang J., Cui G.-S. (2019). Single-cell RNA-seq highlights intra-tumoral heterogeneity and malignant progression in pancreatic ductal adenocarcinoma. Cell Res..

[14] Hiraoka N., Ino Y., Yamazaki-Itoh R., Kanai Y., Kosuge T., Shimada K. (2015). Intratumoral tertiary lymphoid organ is a fa-vourable prognosticator in patients with pancreatic cancer. Br. J. Cancer.

[15] Castino G.F., Cortese N., Capretti G., Serio S., Di Caro G., Mineri R., Magrini E., Grizzi F., Cappello P., Novelli F. (2016). Spatial distribution of B cells predicts prognosis in human pancreatic adenocarcinoma. OncoImmunology.

[16] Gunderson A.J., Rajamanickam V., Bui C., Bernard B., Pucilowska J., Ballesteros-Merino C., Schmidt M., McCarty K., Philips M., Piening B. (2021). Germinal center reactions in tertiary lymphoid structures associate with neoantigen burden, humoral immunity and long-term survivorship in pancreatic cancer. Oncoimmunology.

[17] Zheng L., Ding D., Edil B.H., Judkins C., Durham J.N., Thomas D.L.N., Bever K.M., Mo G., Solt S.E., Hoare J.A. (2021). Vaccine-Induced Intratumoral Lymphoid Aggregates Correlate with Survival Following Treatment with a Neoadjuvant and Adjuvant Vaccine in Patients with Resectable Pancreatic Adenocarcinoma. Clin. Cancer Res..

[18] Tanaka T., Masuda A., Inoue J., Hamada T., Ikegawa T., Toyama H., Sofue K., Shiomi H., Sakai A., Kobayashi T. (2023). Integrated analysis of tertiary lymphoid structures in relation to tumor-infiltrating lymphocytes and patient survival in pan-creatic ductal adenocarcinoma. J. Gastroenterol..

[19] Zou X., Lin X., Cheng H., Chen Y., Wang R., Ma M., Liu Y., Dai Z., Tasiheng Y., Yan Y. (2023). Characterization of in-tratumoral tertiary lymphoid structures in pancreatic ductal adenocarcinoma: Cellular properties and prognostic significance. J. Immunother. Cancer.

[20] Lehmann J., Thelen M., Kreer C., Schran S., Garcia-Marquez M.A., Cisic I., Siepmann K., Hagen E.M., Eckel H.N.C., Lohneis P. (2025). Tertiary Lymphoid Structures in Pancreatic Cancer are Structurally Homologous, Share Gene Expression Patterns and B-cell Clones with Secondary Lymphoid Organs, but Show Increased T-cell Activation. Cancer Immunol. Res..

[21] Hwang W.L., Jagadeesh K.A., Guo J.A., Hoffman H.I., Yadollahpour P., Reeves J.W., Mohan R., Drokhlyansky E., Van Wittenberghe N., Ashenberg O. (2022). Single-nucleus and spatial transcriptome profiling of pancreatic cancer identifies multicellular dynamics associated with neoadjuvant treatment. Nat. Genet..

[22] Sidiropoulos D.N., Shin S.M., Wetzel M., Girgis A.A., Bergman D., Danilova L., Perikala S., Shu D., Montagne J.M., Deshpande A. (2025). Neoadjuvant Immunotherapy Promotes the Formation of Mature Tertiary Lymphoid Structures in a Remodeled Pancreatic Tumor Microenvironment. Cancer Immunol. Res..

[23] Kirschstein E., Harder O., Krull J., Sikorski M., Khanal S., Mack M., Ware C.F., Evans E., Gough M.J., Ma Q. (2026). Myofibroblast programming blocks differentiation of TLS-organizing fibroblastic reticular cells in pancreatic cancer. Cancer Cell.

[24] Satija R., Farrell J.A., Gennert D., Schier A.F., Regev A. (2015). Spatial reconstruction of single-cell gene expression data. Nat. Biotechnol..

[25] Butler A., Hoffman P., Smibert P., Papalexi E., Satija R. (2018). Integrating single-cell transcriptomic data across different condi-tions, technologies, and species. Nat. Biotechnol..

[26] Page M.J., McKenzie J.E., Bossuyt P.M., Boutron I., Hoffmann T.C., Mulrow C.D., Shamseer L., Tetzlaff J.M., Akl E.A., Brennan S.E. (2021). The PRISMA 2020 statement: An updated guideline for reporting systematic reviews. BMJ.

[27] Rethlefsen M.L., Kirtley S., Waffenschmidt S., Ayala A.P., Moher D., Page M.J., Koffel J.B., PRISMA-S Group (2021). PRISMA-S: An extension to the PRISMA Statement for Reporting Literature Searches in Systematic Reviews. Syst. Rev..

[28] Hayden J.A., van der Windt D.A., Cartwright J.L., Côté P., Bombardier C. (2013). Assessing Bias in Studies of Prognostic Factors. Ann. Intern. Med..

[29] Hartung J., Knapp G. (2001). On tests of the overall treatment effect in meta-analysis with normally distributed responses. Stat. Med..

[30] IntHout J., Ioannidis J.P.A., Borm G.F. (2014). The Hartung-Knapp-Sidik-Jonkman method for random effects meta-analysis is straightforward and considerably outperforms the standard DerSimonian-Laird method. BMC Med. Res. Methodol..

[31] Benjamini Y., Hochberg Y. (1995). Controlling the False Discovery Rate: A Practical and Powerful Approach to Multiple Testing. J. R. Stat. Soc. Ser. B Stat. Methodol..

[32] Efron B. (1979). Bootstrap Methods: Another Look at the Jackknife. Ann. Stat..

[33] Fisher R.A. (1925). Statistical Methods for Research Workers.

[34] Zimmerman K.D., Espeland M.A., Langefeld C.D. (2021). A practical solution to pseudoreplication bias in single-cell studies. Nat. Commun..

[35] Squair J.W., Gautier M., Kathe C., Anderson M.A., James N.D., Hutson T.H., Hudelle R., Qaiser T., Matson K.J.E., Barraud Q. (2021). Confronting false discoveries in single-cell differential expression. Nat. Commun..

[36] Cable D.M., Murray E., Zou L.S., Goeva A., Macosko E.Z., Chen F., Irizarry R.A. (2022). Robust decomposition of cell type mixtures in spatial transcriptomics. Nat. Biotechnol..

[37] Nabeya M., Nakajima M., Tsunedomi R., Ozasa T., Kimura Y., Matsui H., Tokumitsu Y., Shindo Y., Iida M., Nakagami Y. (2026). Immature Peritumoral Tertiary Lymphoid Structures Indicate Poor Prognosis in Pancreatic Ductal Adenocarcinoma After NAC. Anticancer Res..

[38] Hu L., Xiao Y., Jiang N., Hu Y., Qiu L., Geng B. (2025). Investigating the correlation between tertiary lymphoid structures and clinical outcomes in pancreatic ductal adenocarcinoma: Insights into tumor immunology. Front. Oncol..

[39] Wang M., Zhang L., Chen H. (2025). Heterogeneity of Tertiary Lymphoid Structures and Plasma Cells in PDAC with and Without Lymph Node Metastasis. Cancers.

[40] Yan Y., Zou X., Lin X., Wang X., Chen Y., Ma M., Wang X., Pan M., Yu X., Liu C. (2025). Assessment of ter-tiary lymphoid structures via 18F-FDG PET/CT scan in patients with pancreatic adenocarcinoma. Cancer Immunol. Immunother..

[41] Chen X. (2024). Study on the Significance of Novel Immune Checkpoints and Immune Architecture Patterns in Pancreatic Ductal Adenocarcinoma. Ph.D. Thesis.

[42] Heumann T., Judkins C., Li K., Lim S.J., Hoare J., Parkinson R., Cao H., Zhang T., Gai J., Celiker B. (2023). A platform trial of neoadjuvant and adjuvant antitumor vaccination alone or in combination with PD-1 antagonist and CD137 agonist anti-bodies in patients with resectable pancreatic adenocarcinoma. Nat. Commun..

[43] Ahmed A., Köhler S., Klotz R., Giese N., Hackert T., Springfeld C., Zörnig I., Jäger D., Halama N. (2022). Tertiary lymphoid structures and their association to immune phenotypes and circulatory IL2 levels in pancreatic ductal adenocarcinoma. On-coimmunology.

[44] Kiryu S., Ito Z., Suka M., Bito T., Kan S., Uchiyama K., Saruta M., Hata T., Takano Y., Fujioka S. (2021). Prognostic value of immune factors in the tumor microenvironment of patients with pancreatic ductal adenocarcinoma. BMC Cancer.

[45] Liudahl S.M., Betts C.B., Sivagnanam S., Morales-Oyarvide V., Da Silva A., Yuan C., Hwang S., Grossblatt-Wait A., Leis K.R., Larson W. (2021). Leukocyte Heterogeneity in Pancreatic Ductal Adenocarcinoma: Phenotypic and Spatial Features Associated with Clinical Outcome. Cancer Discov..

[46] Kuwabara S., Tsuchikawa T., Nakamura T., Hatanaka Y., Hatanaka K.C., Sasaki K., Ono M., Umemoto K., Suzuki T., Sato O. (2019). Prognostic relevance of tertiary lymphoid organs following neoadjuvant chemoradiotherapy in pancreatic ductal adenocarcinoma. Cancer Sci..

[47] Masugi Y., Abe T., Ueno A., Fujii-Nishimura Y., Ojima H., Endo Y., Fujita Y., Kitago M., Shinoda M., Kita-gawa Y. (2019). Characterization of spatial distribution of tumor-infiltrating CD8+ T cells refines their prognostic utility for pancreatic cancer survival. Mod. Pathol..

[48] Wartenberg M., Cibin S., Zlobec I., Vassella E., Eppenberger-Castori S., Terracciano L., Eichmann M.D., Worni M., Gloor B., Perren A. (2018). Integrated Genomic and Immunophenotypic Classification of Pancreatic Can-cer Reveals Three Distinct Subtypes with Prognostic/Predictive Significance. Clin. Cancer Res..

[49] Stromnes I.M., Hulbert A., Pierce R.H., Greenberg P.D., Hingorani S.R. (2017). T-cell Localization, Activation, and Clonal Expansion in Human Pancreatic Ductal Adenocarcinoma. Cancer Immunol. Res..

[50] Diana A., Wang L.M., D’Costa Z., Allen P., Azad A., Silva M.A., Soonawalla Z., Liu S., McKenna W.G., Muschel R.J. (2016). Prognostic value, localization and correlation of PD-1/PD-L1, CD8 and FOXP3 with the desmoplastic stroma in pancreatic ductal adenocarcinoma. Oncotarget.

[51] Poschke I., Faryna M., Bergmann F., Flossdorf M., Lauenstein C., Hermes J., Hinz U., Hank T., Ehrenberg R., Volkmar M. (2016). Identification of a tumor-reactive T-cell repertoire in the immune infiltrate of patients with re-sectable pancreatic ductal adenocarcinoma. Oncoimmunology.

[52] Aziz H.M., Saida L., de Koning W., Stubbs A.P., Li Y., Sideras K., Palacios E., Feliu J., Mendiola M., van Eijck C.H.J. (2023). Spatial genomics reveals a high number and specific location of B cells in the pancreatic ductal adenocarcinoma microen-vironment of long-term survivors. Front. Immunol..

