# Supplementary material for: Prognostic and Spatial Organization of Tertiary Lymphoid Structures in Pancreatic Ductal Adenocarcinoma
Source: Biomedicines. 2026 Oct 9;14(10):2287. doi: 10.3390/biomedicines14102287 (PMC13701594; doi:10.3390/biomedicines14102287)
Supplement: Supplementary file 1 [file biomedicines-14-02287-s001.zip › 01_Methods/Supplementary_Method_S1.pdf]

Supplementary Method S1. Complete database search strategies

The following database-specific strategies correspond to clinical review searches through 25 July 2026. The recorded yields are reported below.

Common concept A — PDAC:

("pancreatic ductal adenocarcinoma" OR "pancreatic ductal adenocarcinomas" OR "pancreatic adenocarcinoma" OR "pancreatic adenocarcinomas" OR "pancreatic cancer" OR "pancreatic cancers" OR "pancreatic neoplasm" OR "pancreatic neoplasms" OR PDAC)

Common concept B — TLS:

("tertiary lymphoid" OR "tertiary lymphoid structure" OR "tertiary lymphoid structures" OR "tertiary lymphoid organ" OR "tertiary lymphoid organs" OR "tertiary lymphoid tissue" OR "tertiary lymphoid tissues" OR "tumor-associated lymphoid structure" OR "tumor-associated lymphoid structures" OR "tumour-associated lymphoid structure" OR "tumour-associated lymphoid structures" OR "ectopic lymphoid structure" OR "ectopic lymphoid structures" OR "ectopic lymphoid organ" OR "ectopic lymphoid organs" OR "lymphoid aggregate" OR "lymphoid aggregates" OR "lymphoid follicle" OR "lymphoid follicles" OR "germinal center" OR "germinal centers" OR "germinal centre" OR "germinal centres" OR TLS OR TLO OR TLT)

PubMed/MEDLINE

Database: PubMed/MEDLINE; coverage from database inception through 25 July 2026; no language or publication-type limits; record yield = 119.

```
(
  "Carcinoma, Pancreatic Ductal"[Mesh]
  OR "Pancreatic Neoplasms"[Mesh]
  OR "pancreatic ductal adenocarcinoma"[Title/Abstract]
  OR "pancreatic ductal adenocarcinomas"[Title/Abstract]
  OR "pancreatic adenocarcinoma"[Title/Abstract]
```

```
  OR "pancreatic adenocarcinomas"[Title/Abstract]
  OR "pancreatic cancer"[Title/Abstract]
  OR "pancreatic cancers"[Title/Abstract]
  OR "pancreatic neoplasm"[Title/Abstract]
  OR "pancreatic neoplasms"[Title/Abstract]
  OR PDAC[Title/Abstract]
)
AND
(
  "Tertiary Lymphoid Structures"[Mesh]
  OR "tertiary lymphoid"[Title/Abstract]
  OR "tertiary lymphoid structure"[Title/Abstract]
  OR "tertiary lymphoid structures"[Title/Abstract]
  OR "tertiary lymphoid organ"[Title/Abstract]
  OR "tertiary lymphoid organs"[Title/Abstract]
  OR "tertiary lymphoid tissue"[Title/Abstract]
  OR "tertiary lymphoid tissues"[Title/Abstract]
  OR "tumor-associated lymphoid structure"[Title/Abstract]
  OR "tumor-associated lymphoid structures"[Title/Abstract]
  OR "tumour-associated lymphoid structure"[Title/Abstract]
  OR "tumour-associated lymphoid structures"[Title/Abstract]
  OR "ectopic lymphoid structure"[Title/Abstract]
  OR "ectopic lymphoid structures"[Title/Abstract]
  OR "ectopic lymphoid organ"[Title/Abstract]
  OR "ectopic lymphoid organs"[Title/Abstract]
  OR "lymphoid aggregate"[Title/Abstract]
  OR "lymphoid aggregates"[Title/Abstract]
  OR "lymphoid follicle"[Title/Abstract]
  OR "lymphoid follicles"[Title/Abstract]
```

  OR "germinal center"[Title/Abstract]

  OR "germinal centers"[Title/Abstract]

  OR "germinal centre"[Title/Abstract]

  OR "germinal centres"[Title/Abstract]

  OR TLS[Title/Abstract]

  OR TLO[Title/Abstract]

  OR TLT[Title/Abstract]

)

Embase.com / Elsevier Emtree interface

Database interface and field syntax: Embase.com, title/abstract/keyword free-text fields; no language or publication-type limit. The final author-verified strategy used the title/abstract/keyword syntax reproduced below.

(

  'pancreatic ductal adenocarcinoma':ti,ab,kw OR

  'pancreatic ductal adenocarcinomas':ti,ab,kw OR

  'pancreatic adenocarcinoma':ti,ab,kw OR

  'pancreatic adenocarcinomas':ti,ab,kw OR

  'pancreatic cancer':ti,ab,kw OR

  'pancreatic cancers':ti,ab,kw OR

  'pancreatic neoplasm':ti,ab,kw OR

  'pancreatic neoplasms':ti,ab,kw OR

  pdac:ti,ab,kw

)

AND

(

  'tertiary lymphoid':ti,ab,kw OR

  'tertiary lymphoid structure':ti,ab,kw OR

  'tertiary lymphoid structures':ti,ab,kw OR

'tertiary lymphoid organ':ti,ab,kw OR

'tertiary lymphoid organs':ti,ab,kw OR

'tertiary lymphoid tissue':ti,ab,kw OR

'tertiary lymphoid tissues':ti,ab,kw OR

'tumor-associated lymphoid structure':ti,ab,kw OR

'tumor-associated lymphoid structures':ti,ab,kw OR

'tumour-associated lymphoid structure':ti,ab,kw OR

'tumour-associated lymphoid structures':ti,ab,kw OR

'ectopic lymphoid structure':ti,ab,kw OR

'ectopic lymphoid structures':ti,ab,kw OR

'ectopic lymphoid organ':ti,ab,kw OR

'ectopic lymphoid organs':ti,ab,kw OR

'lymphoid aggregate':ti,ab,kw OR

'lymphoid aggregates':ti,ab,kw OR

'lymphoid follicle':ti,ab,kw OR

'lymphoid follicles':ti,ab,kw OR

'germinal center':ti,ab,kw OR

'germinal centers':ti,ab,kw OR

'germinal centre':ti,ab,kw OR

'germinal centres':ti,ab,kw OR

tls:ti,ab,kw OR tlo:ti,ab,kw OR tlt:ti,ab,kw

)

Scopus Advanced Search

TITLE-ABS-KEY(

"pancreatic ductal adenocarcinoma" OR

"pancreatic ductal adenocarcinomas" OR

"pancreatic adenocarcinoma" OR

"pancreatic adenocarcinomas" OR

"pancreatic cancer" OR

"pancreatic cancers" OR

"pancreatic neoplasm" OR

"pancreatic neoplasms" OR PDAC

)

AND

TITLE-ABS-KEY(

"tertiary lymphoid" OR

"tertiary lymphoid structure" OR

"tertiary lymphoid structures" OR

"tertiary lymphoid organ" OR

"tertiary lymphoid organs" OR

"tertiary lymphoid tissue" OR

"tertiary lymphoid tissues" OR

"tumor-associated lymphoid structure" OR

"tumor-associated lymphoid structures" OR

"tumour-associated lymphoid structure" OR

"tumour-associated lymphoid structures" OR

"ectopic lymphoid structure" OR

"ectopic lymphoid structures" OR

"ectopic lymphoid organ" OR

"ectopic lymphoid organs" OR

"lymphoid aggregate" OR

"lymphoid aggregates" OR

"lymphoid follicle" OR

"lymphoid follicles" OR

"germinal center" OR

"germinal centers" OR

"germinal centre" OR

"germinal centres" OR TLS OR TLO OR TLT

)

Web of Science Core Collection Advanced Search

TS=(

"pancreatic ductal adenocarcinoma" OR

"pancreatic ductal adenocarcinomas" OR

"pancreatic adenocarcinoma" OR

"pancreatic adenocarcinomas" OR

"pancreatic cancer" OR

"pancreatic cancers" OR

"pancreatic neoplasm" OR

"pancreatic neoplasms" OR PDAC

)

AND

TS=(

"tertiary lymphoid" OR

"tertiary lymphoid structure" OR

"tertiary lymphoid structures" OR

"tertiary lymphoid organ" OR

"tertiary lymphoid organs" OR

"tertiary lymphoid tissue" OR

"tertiary lymphoid tissues" OR

"tumor-associated lymphoid structure" OR

"tumor-associated lymphoid structures" OR

"tumour-associated lymphoid structure" OR

"tumour-associated lymphoid structures" OR

"ectopic lymphoid structure" OR

"ectopic lymphoid structures" OR

"ectopic lymphoid organ" OR

"ectopic lymphoid organs" OR

"lymphoid aggregate" OR

"lymphoid aggregates" OR

"lymphoid follicle" OR

"lymphoid follicles" OR

"germinal center" OR

"germinal centers" OR

"germinal centre" OR

"germinal centres" OR TLS OR TLO OR TLT

)

Wanfang Advanced Search

Database interface and field syntax: Wanfang Knowledge Service Platform advanced search, field = 主题 (topic), with fuzzy matching and English and Chinese free-text terms. The final author-verified query is reproduced below.

主题=("pancreatic ductal adenocarcinoma" OR "pancreatic adenocarcinoma" OR "pancreatic cancer" OR "pancreatic neoplasm" OR PDAC OR 胰腺导管腺癌 OR 胰腺癌)

AND

主题=("tertiary lymphoid" OR "tertiary lymphoid structure" OR "tertiary lymphoid structures" OR "ectopic lymphoid structure" OR "lymphoid aggregate" OR "lymphoid follicle" OR "germinal center" OR TLS OR TLO OR TLT OR 三级淋巴结构 OR 三级淋巴组织 OR 异位淋巴结构 OR 淋巴聚集体 OR 淋巴滤泡 OR 生发中心)

Search limits and recorded yields

- Coverage: database inception to 25 July 2026.

- Language limit: none.

- Publication-type limit: none.

- Record yield: PubMed/MEDLINE 119; Embase 367; Scopus 326; Web of Science Core Collection 215; Wanfang 161.

- The review team retained the available search documentation; the complete author-verified database-specific strings and recorded yields are reproduced here to permit replication.

Gray-literature procedure

No formal gray-literature search was conducted. Gray-literature databases, preprint servers, conference-proceeding databases, and trial registries were not searched. Additional records were sought through reference-list checking of included reports and relevant reviews and through forward citation tracking.
