# Supplementary material for: Prognostic and Spatial Organization of Tertiary Lymphoid Structures in Pancreatic Ductal Adenocarcinoma
Source: Biomedicines. 2026 Oct 9;14(10):2287. doi: 10.3390/biomedicines14102287 (PMC13701594; doi:10.3390/biomedicines14102287)
Supplement: Supplementary file 1 [file biomedicines-14-02287-s001.zip › 01_Methods/Supplementary_Method_S2.pdf]

Supplementary Method S2. Hypothesis-guided framework, fixed program definitions, and analytic eligibility rules

Scope and status

This supplement documents the six internal biological questions, gene-program definitions, scoring rules, and dataset-specific eligibility criteria used in the harmonized secondary reanalysis. The shared configuration (`00_config/00_project_config.R`) fixes the eight TLS programs and three chemokine axes used for implementation. Earlier configuration versions used the label "frozen"; that historical label is not evidence of prospective prespecification. The six-hypothesis framework and program definitions were not prospectively registered before inspection of all contributing molecular datasets and are therefore treated as hypothesis-guided rather than confirmatory. A dated decision record (16 August 2026) documents the GSE202051 composition family/model and the 50-cell compartment eligibility threshold before those models were fitted. The eight-specimens-per-group rule is specified in the retained scoring script, not that dated record. The independent unit was the patient or specimen whenever donor identity was recoverable; datasets without recoverable donor mapping were not used for population-level inference.

S2.1 Hypothesis-guided biological questions

ID	Biological question and expected direction	Primary dataset/contrast	Independent unit	Interpretation boundary
H1	Mature/invasive-border TLS regions have a higher mature-TLS score than TLS-zero regions.	GSE277116; pathology TLS-positive minus TLS-zero spots	Patient after within-patient section aggregation	Spatial association only; no causal TLS effect.
H2	B-cell, GC-B, Tfh/CXCL13, and FDC/HEV programs co-vary with pathology TLS burden.	GSE277116; within-patient TLS percentage correlations	Tumor patient	Co-enrichment only; not a cell–cell communication network.
H3	rCAF-organizer programs align more closely with lymphoid/TLS measures than myCAF/TGFβ programs.	GSE277116 and GSE322767	Patient	No lineage conversion, fibroblast causality, or recruitment claim.
H4	Treatment context is associated with specimen-level tissue composition and compartment-specific programs.	GSE202051; treated versus untreated	Specimen (`pid`)	Unpaired observational comparison; not treatment induction.
H5	CXCL13–CXCR5, CCL19/CCL21–CCR7, and LTB–LTBR show spatial co-enrichment in TLS-positive tumor niches.	GSE277116	Tumor patient	Co-expression does not prove ligand–receptor binding or signalling.
H6	Long-term recurrence-free tumors have a higher mature-TLS targeted-panel score.	GSE220929; long versus short recurrence-free group	Patient/sample	Exploratory targeted-panel association; not a validated predictor.

S2.2 Fixed gene-program definitions

The programs below are internally defined marker sets assembled from the cited TLS and CAF literature; they are not proprietary published signatures. Their gene membership is fixed in the shared analysis configuration for reproducible implementation. This fixed implementation should not be interpreted as evidence that the programs were prospectively specified before all datasets were inspected. Scoring is analysis-specific rather than one universal formula; the applicable expression scale, standardization axis, and aggregation unit are specified in S2.2.1. No gene was added in response to the observed outcome within a given analysis.

Program	Gene membership	Biological/source rationale
B cell	CD79A; MS4A1; CD74; HLA-DRA	Canonical B-cell and antigen-presentation markers for TLS lymphoid compartments [1,2,16,20].
GC B	AICDA; BCL6; MEF2B; RGS13	Germinal-centre B-cell differentiation/activity markers associated with mature TLS [1,2,16,20].
Tfh/CXCL13	CXCL13; PDCD1; ICOS; CXCR5; SH2D1A	Tfh and CXCL13-axis markers supporting B-cell recruitment and germinal-centre organization [1,2,16,20].
Plasma cell	MZB1; JCHAIN; XBP1; SDC1; DERL3	Antibody-secreting-cell program, a feature of functionally mature TLS [1,2,16,20].
FDC/HEV	CXCL13; CCL21; CLDN5; SELP; ACKR1	FDC/HEV organizer and vascular markers supporting structured TLS niches [1,2,16,20].
rCAF organizer	CCL21; CCL19; CCL13; CXCL12; COL3A1	Reticular/lymphoid-organizer fibroblast program assembled from TLS and CAF biology [4–10,23].
myCAF/TGFβ	ACTA2; TAGLN; MYL9; COL1A1; TGFB1	Contractile, matrix-rich myofibroblast/TGFβ stromal program [4–10,23].
Mature TLS	CD79A; MS4A1; CXCL13; CCL21; MZB1; JCHAIN; AICDA; BCL6	Composite spanning B-cell, germinal-centre, chemokine/organizer, and plasma-cell features [1,2,16,18–20].

S2.2.1 Score construction and TLS-state coding

GSE277116 Visium spot scores (Figures 5–6): raw counts were converted within each spot to log1p(CP10k), where CP10k = gene count / total spot counts × 10,000. For each program gene, its log1p(CP10k) values were z-standardized across spots within each tissue section; non-finite values (including zero-variance genes) were set to zero. The spot score was the arithmetic mean across the program genes present in the expression matrix. Section-level estimates were then aggregated within patient for inferential summaries; spots and sections were not treated as independent patients.

Figure 6 TLS states were coded directly from the pathology annotation: TLS-zero = TLS_pct 0; TLS-core = TLS_pct 100; TLS-mixed = 0 < TLS_pct < 100. The continuous TLS_pct measure was retained for the primary within-patient Spearman analyses.

The four score-method sensitivity variants used the same spots and genes: (1) mean gene-wise z-scored log1p(CP10k) across spots within section (primary); (2) mean log1p(CP10k); (3) mean CP10k; and (4) fraction of program genes detected (raw count > 0). Sensitivity was assessed by patient-level Spearman correlation with TLS_pct.

GSE202051 compartment pseudobulk scores: the retained downstream analysis starts from the supplied specimen-by-compartment logCPM table (GSE202051_tls_pseudobulk_logcpm.csv). The dated GSE202051 decision record describes upstream pseudobulk construction by summing the deposited integer counts within pid and biological compartment, followed by logCPM transformation; the compact supplied workflow does not independently rebuild that upstream object. For scoring, each gene's supplied logCPM values were z-standardized across eligible specimens within the assigned compartment. Zero-variance or non-finite gene scores were omitted, and the program score was the arithmetic mean of the remaining gene scores. A compartment required at least 50 cells per eligible specimen, and a treatment contrast required at least eight eligible specimens per group. The retained analysis fits equal-specimen-weight linear models and adjusts all eight program P values jointly by BH. These are specimen-level program scores, not cell- or spot-level independent observations.

Other external datasets used their script-specific transformations rather than the Visium formula: GSE220929 used log2 library-size-normalized counts with a 0.5 pseudocount, followed by gene-wise z-scoring across samples; GSE129492 averaged the available deposited expression values without additional transformation or z-scoring; GSE225767 used log2(count + 1), followed by gene-wise z-scoring across samples. Targeted panels required ≥3 of 8 mature-TLS genes for GSE220929/GSE226840, whereas the GSE225767 script required all eight. GSE240078 was explicitly a two-gene B/GC proxy, not the full mature-TLS score.

S2.3 Dataset-specific availability and eligibility rules

Analysis	Platform/data state	Rule implemented	Evidence/output
GSE202051 compartment pseudobulk	Whole-transcriptome single-nucleus data	A specimen required at least 50 cells in the biologically assigned compartment. A program comparison required at least 8 eligible specimens in each treatment group. Gene-wise zero cross-specimen variance was excluded before averaging standardized logCPM.	`00_config/GSE202051_FIGURE4_COMPOSITION_DECISIONS_2026-08-16.md`; `02_analyze/05_gse202051_tls_pseudobulk.R`
GSE202051 composition sensitivity	Deposited cell/nucleus composition	Primary analysis retained all 43 `pid` values. The sensitivity analysis excluded specimens with fewer than 2,000 deposited cells; it is not a primary-exclusion rule.	`02_analyze/06_gse202051_composition_sensitivity.R`
GSE202051 program availability	Whole-transcriptome single-nucleus data	All 31 unique genes used across the eight fixed programs were present in the availability table.	`results/tables/GSE202051_tls_program_gene_coverage.csv`

Analysis	Platform/data state	Rule implemented	Evidence/output
GSE277116 spatial program/axis analyses	Visium sections with pathology TLS annotation	Effects were first calculated within section and aggregated within patient; section-level values were descriptive. Chemokine-axis coverage was checked in every section before analysis.	00_config/UPSTREAM_PROVENANCE.md; 02_analyze/05_patient_level_tls.R; 02_analyze/08_chemokine_axes.R
GSE220929 targeted panel	Deposited targeted count matrix	The mature-TLS panel required at least 3 of 8 requested genes; otherwise the script stops. Ten long- and ten short-recurrence-free samples were analysed.	`02_analyze/09_gse220929_targeted_panel.R`
GSE226840 targeted panel	NanoString RCC files	The mature-TLS panel required at least 3 of 8 requested genes for score construction. Fourteen TLS microdissections were derived from tumors from eight patients, but the region-to-patient mapping was unavailable; therefore no donor-aware inferential maturation test was performed.	02_analyze/10_gse226840_rcc_maturation.R; results/tables/WP0_GSE226840_geo_samples.csv; retained only as a dataset-level audit.
GSE225767 post-SBRT panel	Deposited count matrix	All 8 mature-TLS genes were required (stopifnot); 20 post-SBRT PDAC samples were analysed (15 responders, 5 non-responders).	02_analyze/19_gse225767_post_sbrt_tls_response.R; results/tables/Figure9_GSE225767_postSBRT_TLS_response.csv
GSE240078 stromal proxy	Spatially resolved stromal expression with patient identifiers	Stromal measurements were aggregated within patient. Only the available two-gene B/GC proxy was analysed; it was not represented as the full mature-TLS program. The treated-versus-untreated comparison was exploratory.	02_analyze/14_gse240078_patient_stroma_tls_program.R; results/tables/External_GSE240078_patient_stroma_test.csv

GSE202051 composition analysis: all 43 specimens (18 untreated, 25 treated) and all 12 Level 1 populations were retained in the primary analysis. Each population was analyzed with an equal-specimen-weight quasi-binomial logit model of specimen-level proportion by treatment; the 12 population tests formed one BH family. No standard-error-based category exclusion was implemented. The separate sensitivity analysis excluded specimens with fewer than 2,000 nuclei. No universal numeric minimum gene-coverage threshold for every whole-transcriptome analysis was documented; dataset-specific availability rules are listed above and should not be represented as one common prospectively prespecified cutoff.

S2.4 Stability, testing, and multiplicity rules

- Spatial analyses used patient-level aggregation. Section, spot, nucleus, and region counts were not treated as independent patient counts.
- For small spatial cohorts, two-sided exact sign-flip inference enumerated the possible patient-sign assignments; bootstrap confidence intervals resampled the patient/specimen unit. For Figures 6B and 7B, the descriptive point estimate is the mean patient Fisher-z correlation back-transformed to rho. The pointwise 95% interval is the percentile interval of the same back-transformed statistic over 10,000 patient resamples, rather than an interval for the arithmetic mean of raw rho. The updated reconciliation uses the supplied

patient-correlation tables with program-specific fixed seeds recorded in each result table; it does not rebuild spot-level correlations. Exact sign-flip tests and the directional binomial sign sensitivity use raw patient correlations and retain their original seven-program (Figure 6) and six-program (Figure 7) BH families. The tables label arithmetic_mean_rho separately; mean_rho, ci_lower, and ci_upper describe the displayed Fisher-z summary. Pointwise confidence intervals are not multiplicity-adjusted. The portable implementation is 02_analyze/correlation_summary_scaling.py; Figure 6B/7B are rendered from its summaries by 03_plot/correlation_panels_compose.py.

- Benjamini–Hochberg correction was applied within families grouping comparisons by dataset, estimand, biological level, and analysis type. The dated GSE202051 record documents the 12-population composition family before its raw-object models were fitted. For other analyses, a grouping rationale does not establish temporal prespecification. The principal families and their sizes are summarized in Table S4. Within-family corrections do not control the study-wide false-discovery rate, and the three external-dataset tests retain cohort-specific nominal P values without a cross-dataset correction. Secondary, component, sensitivity, and cross-dataset findings are interpreted as hypothesis-guided or exploratory and are not treated as independent validation. For GSE202051, the 12 cell-composition tests and the eight compartment-aware TLS-program treatment contrasts are separate BH families: the former assess specimen-level cell fractions, whereas the latter assess program scores in their assigned biological compartments. The retained pseudobulk script applies BH jointly to all eight program P values, rather than separately within each compartment.
- Estimability was determined by the explicit dataset-specific rules in S2.3 and successful computation of the stated model or test. No additional universal numeric “stability” or standard-error cutoff was applied. Where a script required a minimum gene count or sample count, it stopped rather than assigning a null result when that condition was not met.
- The targeted-panel rule of at least three available program genes is an outcome-blind platform amendment. It is not equivalent to whole-transcriptome full-program coverage.
