# Supplementary material for: Prognostic and Spatial Organization of Tertiary Lymphoid Structures in Pancreatic Ductal Adenocarcinoma
Source: Biomedicines. 2026 Oct 9;14(10):2287. doi: 10.3390/biomedicines14102287 (PMC13701594; doi:10.3390/biomedicines14102287)
Supplement: Supplementary file 1 [file biomedicines-14-02287-s001.zip › 01_Methods/Supplementary_Method_S3.pdf]

Supplementary Method S3. Biological rationale for the hypothesis-guided framework

Purpose and interpretation boundary

This table records the biological rationale underlying the six internally defined biological questions and maps each question to the relevant dataset, independent unit, manuscript references, and interpretation boundary. It documents scientific context and does not constitute preregistration or evidence of prospective prespecification. It does not convert spatial association into causal or mechanistic evidence.

Hypothesis	Biological rationale/context	Supporting manuscript references	Analysis and interpretation boundary
H1: mature-TLS enrichment in pathology-defined TLS regions	Mature TLS are expected to contain organized B-cell, T-cell-help, antigen-presentation, and plasma-cell programs; a composite score was used to test whether these features co-occur in pathology-defined TLS niches.	[1,2,16,18–20]	GSE277116, TLS-positive versus TLS-zero spot contrast aggregated by patient. Positive enrichment supports spatial co-localization, not TLS causation or function.
H2: B-cell/GC-B/Tfh/FDC maturation axis	The component programs represent complementary features expected in a mature lymphoid niche: B-cell presence, germinal-centre-associated B-cell activity, Tfh/CXCL13, and FDC/HEV-related organization.	[1,2,16,20]	GSE277116, patient-level correlations with pathology TLS burden. The analysis tests coordinated program variation, not a cell–cell communication network.

Hypothesis	Biological rationale/context	Supporting manuscript references	Analysis and interpretation boundary
H3: rCAF-organizer versus myCAF/TGFβ relationship	PDAC fibroblast states are heterogeneous. The contrast was designed to test whether distinct deposited CAF programs show opposite spatial association with lymphoid probability, rather than assuming all CAF states have one role.	[4–10,23]	GSE277116 and GSE322767, patient-level program associations. No inference is made about fibroblast lineage conversion, TGFβ dependence, or causal recruitment.
H4: treatment-associated remodeling	Treatment-exposed and untreated PDAC specimens may differ in cell composition and microenvironmental programs. The hypothesis was framed as an observational specimen-level comparison because the public cohort is cross-sectional and unpaired.	[21,22]	GSE202051, treated versus untreated specimen-level composition/program analyses. Results do not demonstrate treatment-induced TLS formation.
H5: chemokine-axis co-enrichment	CXCL13–CXCR5, CCL19/CCL21–CCR7, and LTB–LTBR were selected as canonical lymphoid-organization axes to assess repeated spatial co-enrichment in pathology TLS-positive niches.	[1,2,16,20]	GSE277116, patient-level spatial standardized differences. Co-expression does not identify sender/receiver cells, ligand binding, signalling, or causal direction.
H6: mature-TLS score and recurrence-free status	Clinical TLS studies report a favorable prognostic association, providing a bounded reason to test the targeted-panel score defined in the analysis configuration in an independent outcome-labelled cohort.	[14–19]	GSE220929, long versus short recurrence-free groups. This small targeted-panel analysis is exploratory and cannot validate a universal prognostic assay.
