# Supplementary material for: Prognostic and Spatial Organization of Tertiary Lymphoid Structures in Pancreatic Ductal Adenocarcinoma
Source: Biomedicines. 2026 Oct 9;14(10):2287. doi: 10.3390/biomedicines14102287 (PMC13701594; doi:10.3390/biomedicines14102287)
Supplement: Supplementary file 1 [file biomedicines-14-02287-s001.zip › 02_Tables/Table_S1_Clinical_Cohorts_and_OS_Traceability.pdf]

Table S1. Clinical cohort and extraction-status ledger

Report ID	Cohort	Country	Design	Eligible PDAC (n)	TLS evaluable (n)	Outcome evaluable (n)	Treatment context
FT-001	Upfront surgery	Japan	Retrospective surgical cohort	64	64.0	64.0	UPFRONT_OR_TREATMENT_NAIVE
FT-001	Neoadjuvant chemotherapy followed by surgery	Japan	Retrospective post-NAC surgical cohort	67	67.0	67.0	POST_NAC_NAT
FT-002	Cologne/Munich resected treatment-naive cohort	Germany	Retrospective multicentre surgical cohort	110	110.0	74.0	UPFRONT_OR_TREATMENT_NAIVE
FT-003	CAMS/PUMC resected PDAC cohort	China	Retrospective single-institution surgical cohort	282	282.0	282.0	UPFRONT_OR_TREATMENT_NAIVE
FT-004	Surgery-alone cohort	China	Retrospective surgical cohort	380	380.0	380.0	UPFRONT_OR_TREATMENT_NAIVE
FT-004	Neoadjuvant-treated cohort	China	Retrospective post-NAT surgical cohort	136	136.0	136.0	POST_NAC_NAT
FT-005	Kobe University resected PDAC cohort	Japan	Retrospective consecutive surgical cohort	162	162.0	162.0	UPFRONT_OR_TREATMENT_NAIVE
FT-007	Surgery-first stratum	Japan	Retrospective surgical cohort	93	93.0	93.0	UPFRONT_OR_TREATMENT_NAIVE
FT-007	Neoadjuvant chemoradiotherapy stratum	Japan	Retrospective post-NACRT surgical cohort	47	47.0	47.0	POST_NAC_NAT
FT-008	Derivation resected PDC cohort	Japan	Retrospective surgical cohort	308	308.0	308.0	UPFRONT_OR_TREATMENT_NAIVE
FT-008	Independent validation cohort	Japan	Retrospective validation surgical cohort	226	226.0	220.0	UPFRONT_OR_TREATMENT_NAIVE
FT-009	Dalian Medical University cohort	China	Retrospective surgical cohort	69	69.0	69.0	UPFRONT_OR_TREATMENT_NAIVE
FT-010	CQMU pathology cohort	China	Retrospective surgical cohort	65	65.0	65.0	UPFRONT_OR_TREATMENT_NAIVE
FT-010	TCGA-PAAD pathology-classified cohort	United States / multicentre	Public dataset validation cohort	143	143.0	143.0	UPFRONT_OR_TREATMENT_NAIVE
FT-013	Heidelberg resected PDAC cohort	Germany	Retrospective surgical cohort	55	55.0	55.0	UPFRONT_OR_TREATMENT_NAIVE
FT-015	Resectable primary-surgery cohort	United States	Retrospective surgical cohort	63	63.0	63.0	UPFRONT_OR_TREATMENT_NAIVE

Report ID	Cohort	Country	Design	Eligible PDAC (n)	TLS evaluable (n)	Outcome evaluable (n)	Treatment context
FT-016	R0-resected PDAC cohort	Japan	Retrospective surgical cohort	85	85.0	85.0	UPFRONT_OR_TREATMENT_NAIVE
FT-017	GVAX neoadjuvant/adjuvant vaccine cohort	United States	Clinical trial correlative cohort	76	66.0	66.0	INDUCED_VACCINE
FT-018	Resected PDAC cohort	United States	Retrospective surgical cohort	214	214.0	208.0	UPFRONT_OR_TREATMENT_NAIVE
FT-019	Molecularly profiled resected PDAC cohort	Switzerland	Retrospective surgical molecular-pathology cohort	110			UPFRONT_OR_TREATMENT_NAIVE
FT-020	Adjuvant-treated resected PDAC cohort	Germany	Retrospective whole-mount surgical cohort	145	145.0	145.0	UPFRONT_OR_TREATMENT_NAIVE
FT-021	Humanitas non-metastatic resected PDAC cohort	Italy	Retrospective consecutive surgical cohort	104	104.0	104.0	UPFRONT_OR_TREATMENT_NAIVE
FT-027	Resected PDAC cohort	China	Retrospective surgical imaging-pathology cohort	266	266.0	266.0	UPFRONT_OR_TREATMENT_NAIVE
FT-034	GVAX+nivolumab+urelumab platform trial	United States	Platform clinical trial correlative cohort	40	23.0	40.0	INDUCED_IMMUNOTHERAPY
FT-039	Untreated discovery pathology cohort	United States	Retrospective spatial pathology cohort	135	104.0	104.0	UPFRONT_OR_TREATMENT_NAIVE
FT-041	Small resected PDA survival subset	United States	Translational tissue cohort	20	14.0	14.0	MIXED_UNRESOLVED
FT-042	Heidelberg resected PDAC immune cohort	Germany	Retrospective surgical immune-pathology cohort	127			UPFRONT_OR_TREATMENT_NAIVE
FT-004	NAT peri/intratumoral spatial subset	China	Spatial pathology subset	22	22.0	22.0	POST_NAC_NAT
FT-013	Xena public transcriptomic survival cohort	Public dataset	Public transcriptomic survival validation cohort	183	183.0	183.0	PUBLIC_TRANSCRIPTOMIC_DATASET
FT-041	All PDA tissues with TLS classification	United States	Translational mixed-treatment tissue cohort	20	20.0		MIXED_UNRESOLVED

This cohort-level ledger maps the 21 included studies represented by 22 reports. Multiple rows may arise from one report when it contains distinct cohorts, treatment strata, or non-overlapping analytic subsets. Screening, extraction, overlap resolution, and pool membership were independently checked by two reviewers and resolved by consensus.

Primary overall-survival meta-analysis pool

These five independent resection cohorts (total n = 1,019) were pooled because each reported an adjusted Cox estimate for a direct histological TLS-presence-versus-absence contrast. Density, continuous area, maturity-only, expression-signature, and post-neoadjuvant effects were excluded from the primary pool. The intratumoral-only pool was analysed separately.

Effect/report ID	Study/cohort	Analysis n	Original TLS comparison	Original adjusted HR (95% CI)	Adjustment covariates/model	Transformation/harmonization	Final harmonized HR (95% CI)	Effect model	Source locator
E009 / FT-004	Zou et al., 2023 / FT004-SA	380	Intratumoral TLS present vs absent	0.695 (0.528–0.915)	TNM stage, grade, iTLS	None	0.695 (0.528–0.915)	Adjusted Cox HR	PDF p. 9
E012 / FT-005	Tanaka et al., 2023 / FT005-COH	162	Any TLS present vs absent	0.420 (0.280–0.620)	Tumor-characteristic Cox model; backward selection	None	0.420 (0.280–0.620)	Adjusted Cox HR	PDF p. 6
E014 / FT-008	Hiraoka et al., 2015 / FT008-DER	308	Intratumoral TLO absent vs present	1.637 (1.115–2.403)	Size; nodal/distant status; grade; margin; lymphatic, venous, and neural invasion	Reciprocal inversion of HR and CI	0.611 (0.416–0.897)	Adjusted Cox HR	PDF p. 5
E022 / FT-010	Hu et al., 2025 / FT010-CQMU	65	Intratumoral TLS present vs absent	0.407 (0.180–0.921)	Variables with univariable p<0.05; N classification retained	None	0.407 (0.180–0.921)	Adjusted Cox HR	PDF p. 4
E043 / FT-042	Poschke et al., 2016 / FT042-COH	104*	Any TLS present vs absent	0.440 (0.240–0.810)	TLS, grade, metastasis, CA19-9, ASA class; complete-case Cox	None	0.440 (0.240–0.810)	Adjusted Cox HR	Supplement

*Primary OS pool traceability. All five effects were multivariable Cox estimates for a direct binary pathology-defined TLS/TLO comparison. HR < 1 favours TLS presence after harmonization. *Poschke et al.: source cohort n=127; complete-case multivariable model n=104. No other primary-pool estimate required transformation.*
