# Supplementary material for: Prognostic and Spatial Organization of Tertiary Lymphoid Structures in Pancreatic Ductal Adenocarcinoma
Source: Biomedicines. 2026 Oct 9;14(10):2287. doi: 10.3390/biomedicines14102287 (PMC13701594; doi:10.3390/biomedicines14102287)
Supplement: Supplementary file 1 [file biomedicines-14-02287-s001.zip › 02_Tables/Table_S2_Clinical_Association_Effects.pdf]

Table S2. Harmonized clinical association effects and source 2×2 data

Effect ID	Clinical feature	Study	Treatment context	TLS+ (exposure/reference)	TLS− (exposure/reference)	Known-case denominator	OR (95% CI)
X2-021	Female sex	Zou et al., 2023 (FT-004)	Surgery alone	34/46	130/170	380	0.967 (0.587–1.591)
X2-006	Female sex	Poschke et al., 2016 (FT-042)	Mixed treatment	22/29	38/38	127	0.759 (0.372–1.549)
X2-024	Pathologic node positivity	Zou et al., 2023 (FT-004)	Surgery alone	41/39	155/145	380	0.983 (0.6–1.611)
X2-009	Pathologic node positivity	Poschke et al., 2016 (FT-042)	Mixed treatment	41/10	62/14	127	0.926 (0.376–2.282)
X2-025	High histologic grade	Zou et al., 2023 (FT-004)	Surgery alone	29/51	130/170	380	0.744 (0.447–1.238)
X2-011	High histologic grade	Poschke et al., 2016 (FT-042)	Mixed treatment	28/20	33/33	114	1.4 (0.661–2.963)
X2-026	Body/tail tumour location	Zou et al., 2023 (FT-004)	Surgery alone	35/45	136/154	370	0.881 (0.535–1.45)
X2-008	Body/tail tumour location	Poschke et al., 2016 (FT-042)	Mixed treatment	16/35	32/44	127	0.629 (0.298–1.326)
X2-003	Neoadjuvant treatment	Zou et al., 2023 (FT-004)	Post-NAT vs surgery alone	21/80	115/300	516	0.685 (0.404–1.159)
X2-013	Neoadjuvant treatment	Poschke et al., 2016 (FT-042)	Mixed treatment	2/49	12/64	127	0.218 (0.047–1.018)
X2-022	Advanced pathologic stage	Zou et al., 2023 (FT-004)	Surgery alone	21/58	98/199	376	0.735 (0.422–1.28)
X2-043	Advanced pathologic stage	Yan et al., 2025 (FT-027)	Treatment not stratified	20/41	85/120	266	0.689 (0.377–1.258)

All 12 harmonized clinical association effects are shown. No cross-study pooled estimates were calculated for these heterogeneous clinicopathological associations. Source extraction and harmonization were independently checked by two reviewers and resolved by consensus.
