# Supplementary material for: Prognostic and Spatial Organization of Tertiary Lymphoid Structures in Pancreatic Ductal Adenocarcinoma
Source: Biomedicines. 2026 Oct 9;14(10):2287. doi: 10.3390/biomedicines14102287 (PMC13701594; doi:10.3390/biomedicines14102287)
Supplement: Supplementary file 1 [file biomedicines-14-02287-s001.zip › 02_Tables/Table_S3_Analysis_Register.pdf]

Table S3. Analysis claim-to-estimand and reproducibility-status register

Claim ID	Analysis scope	Dataset	Independent unit	Primary estimand	Planned model	Required sensitivity analysis	Current status
C4_01	Treatment-associated cell composition	GSE202051	specimen	treated versus untreated cell-type proportion	quasi-binomial or beta-binomial specimen-level model with treatment as fixed effect	exclude low-quality specimens and report composition/QC balance	Complete; all 43 specimens retained; primary and ≥2,000-cell sensitivity outputs supplied.
C4_02	Treatment-associated TLS programs	GSE202051	specimen	treated versus untreated specimen-level TLS program score	cell-type-aware specimen pseudobulk model or clearly labelled exploratory model	restrict to tumor specimens and report per-cell-type results	Complete; compartment-level analysis supplied; no program passed BH correction.
C5_01	Pathology-defined mature-TLS enrichment assessment	GSE277116	patient	within-patient TLS-positive minus TLS-zero mature-TLS score	patient-level paired/aggregated contrast with bootstrap CI	section aggregation and exclusion of patients C4/C10 (sections J1568_C4/J1568_C7)	Complete; section effects aggregated within patient; tumor-subset sensitivity supplied.
C6_01	TLS cellular ecology	GSE277116	patient	patient-level association of TLS burden with fixed program scores	Within-patient Spearman; descriptive mean Fisher-z and matched patient-bootstrap interval; exact sign-flip tests on raw patient correlations	alternative TLS threshold and section aggregation	Complete; patient-level correlations, score sensitivity, and leave-one-patient-out results supplied.
C7_01	CAF-lymphoid ecology	GSE322767	patient	rCAF versus myCAF within-patient correlation difference with lymphoid probability	paired patient-level correlation contrast and exact/permutation sign test	leave-one-patient-out and score-provenance sensitivity	Complete; exact patient-level contrast and leave-one-patient-out sensitivity supplied.
C8_01	Chemokine axes	GSE277116	patient	within-patient TLS-positive minus TLS-zero score for three hypothesis-guided axes	patient-level standardized contrast with bootstrap CI and BH correction	alternative TLS threshold and gene-coverage report	Complete; gene coverage, patient-level axis effects, and TLS-high sensitivity supplied.

Claim ID	Analysis scope	Dataset	Independent unit	Primary estimand	Planned model	Required sensitivity analysis	Current status
C9_01	Exploratory targeted-panel outcome assessment	GSE220929	patient	Long versus Short mature-TLS targeted-panel score	fixed targeted-panel score contrast with exact/permutation inference	report all measured component genes and no survival-time claim	Complete; exact permutation and component-gene coverage supplied; interpreted as exploratory.
C9_02	GSE226840 dataset audit	GSE226840	No recoverable independent unit for inferential analysis	Gene coverage and CD23-defined TLS-category audit only	No inferential model; region-level P values not reported because 14 TLS regions arose from 8 patients and region-to-patient mapping was unavailable	Confirm public sample count, TLS categories, and absence of donor mapping	Complete; dataset retained for audit only and excluded from inferential cross-dataset assessment.

This register links each molecular-analysis claim to its estimand, independent unit, analysis, sensitivity analysis, and reproducibility output supplied with the revision.
