# Supplementary material for: Prognostic and Spatial Organization of Tertiary Lymphoid Structures in Pancreatic Ductal Adenocarcinoma
Source: Biomedicines. 2026 Oct 9;14(10):2287. doi: 10.3390/biomedicines14102287 (PMC13701594; doi:10.3390/biomedicines14102287)
Supplement: Supplementary file 1 [file biomedicines-14-02287-s001.zip › 02_Tables/Table_S4_Multiplicity_Families.pdf]

Table S4. Multiple-testing families and interpretation boundaries

Testing families group comparisons by dataset, estimand, biological level, and analysis type. BH correction controls multiplicity within the reported family; it does not provide study-wide confirmatory error control or establish that the grouping was prospectively specified. Tissue-scope, component, and sensitivity results remain related analyses rather than independent replications. For the Figure 6B and Figure 7B correlation displays, pointwise patient-bootstrap intervals summarize mean Fisher-z correlations back-transformed to rho; they are not multiplicity-adjusted. The retained exact tests use raw patient correlations, with directional sign tests adjusted separately across the same seven or six programs. These descriptive-scale clarifications do not change family membership or the retained P and BH values.

Dataset / analysis	Testing family	Number of tests	Rationale for family	Interpretation
GSE202051 composition	Level-1 specimen composition	12	Same dataset, treatment contrast, outcome type, and specimen-level estimand	Hypothesis-guided; dated pre-model family record
GSE202051 compartment-aware TLS pseudobulk	Eight program-specific treatment contrasts across assigned biological compartments	8	Same dataset and treatment comparison; BH is applied jointly to all eight program tests in the retained script	Exploratory specimen-level association; dataset-specific dated decision record; no study-wide confirmatory error control
GSE277116 TLS-program enrichment	Fixed TLS programs within each stated tissue scope	8 per reported tissue scope	Same pathology contrast and patient-level estimand within each scope; overall, tumor, and lymph-node scopes are adjusted separately and are not independent replications	Hypothesis-guided; patient-level inference

Dataset / analysis	Testing family	Number of tests	Rationale for family	Interpretation
GSE277116 continuous TLS ecology	Program correlations with TLS burden	7	Same patients, TLS-burden estimand, and correlation analysis	Hypothesis-guided; patient-level summary
GSE322767 CAF ecology	Deposited CAF programs	6	Same patients, lymphoid-probability estimand, and correlation analysis	Hypothesis-guided / secondary
GSE277116 chemokine axes	Composite chemokine axes	3	Same patient-level TLS-positive versus TLS-zero composite-axis estimand	Hypothesis-guided
GSE277116 chemokine components	Component genes	7	Component-level decomposition is distinct from composite-axis inference	Exploratory component analysis
RCTD neighborhood analysis	Eligible cell types	21	Same neighborhood-minus-background patient-level estimand	Exploratory sensitivity analysis
TLS-core distance gradient	Chemokine axes	3	Same post hoc core-minus-far patient-level estimand	Post hoc exploratory
External datasets	One retained contrast per patient-level dataset	1 per dataset; 3 cohort-specific tests in total	Different platforms, endpoints, and gene coverage; no cross-dataset BH adjustment or pooled effect	Nominal exploratory P values; no study-wide error control or validation claim

Dataset / analysis	Testing family	Number of tests	Rationale for family	Interpretation
GSE277116 score-construction sensitivity	TLS-burden correlations within each score definition	7 per score definition; 4 definitions including the primary score	Identical patients and TLS-burden estimand; BH applied separately within each score method in the retained sensitivity script	Related sensitivity results; no independent replication or study-wide error control
GSE322767 direct CAF contrast	Within-patient rCAF-minus-myCAF correlation contrast	1	Single direct contrast, separate from the six-program omnibus family; reported exact P value is nominal	Hypothesis-guided comparison; no additional study-wide correction
