# Supplementary material for: Prognostic and Spatial Organization of Tertiary Lymphoid Structures in Pancreatic Ductal Adenocarcinoma
Source: Biomedicines. 2026 Oct 9;14(10):2287. doi: 10.3390/biomedicines14102287 (PMC13701594; doi:10.3390/biomedicines14102287)

Supplementary Figure S1 | QUIPS risk-of-bias assessment

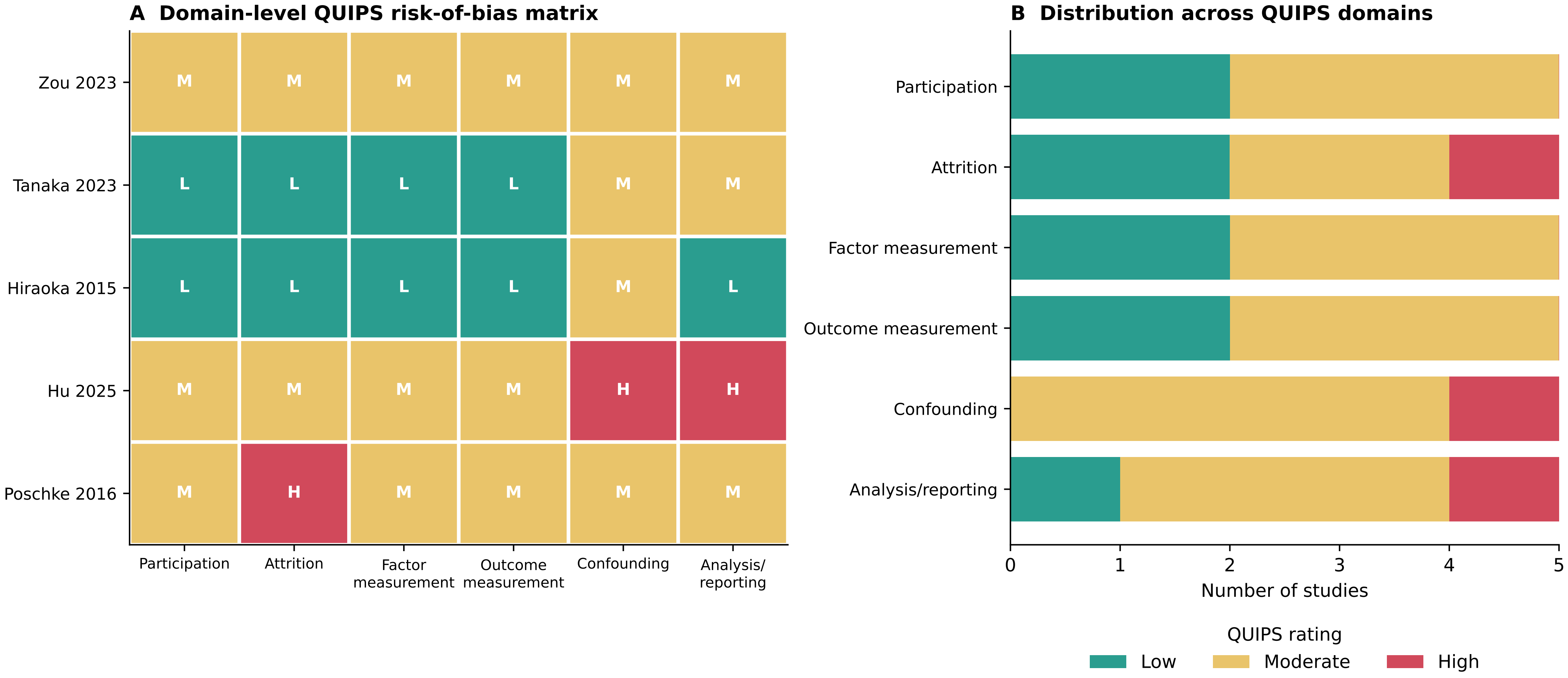

Supplement: Supplementary file 1 [file biomedicines-14-02287-s001.zip › 03_Figures/Figure_S1.pdf]
