# Supplementary material for: Prognostic and Spatial Organization of Tertiary Lymphoid Structures in Pancreatic Ductal Adenocarcinoma
Source: Biomedicines. 2026 Oct 9;14(10):2287. doi: 10.3390/biomedicines14102287 (PMC13701594; doi:10.3390/biomedicines14102287)
Supplement: Supplementary file 1 [file biomedicines-14-02287-s001.zip › 03_Figures/Figure_S2.pdf]

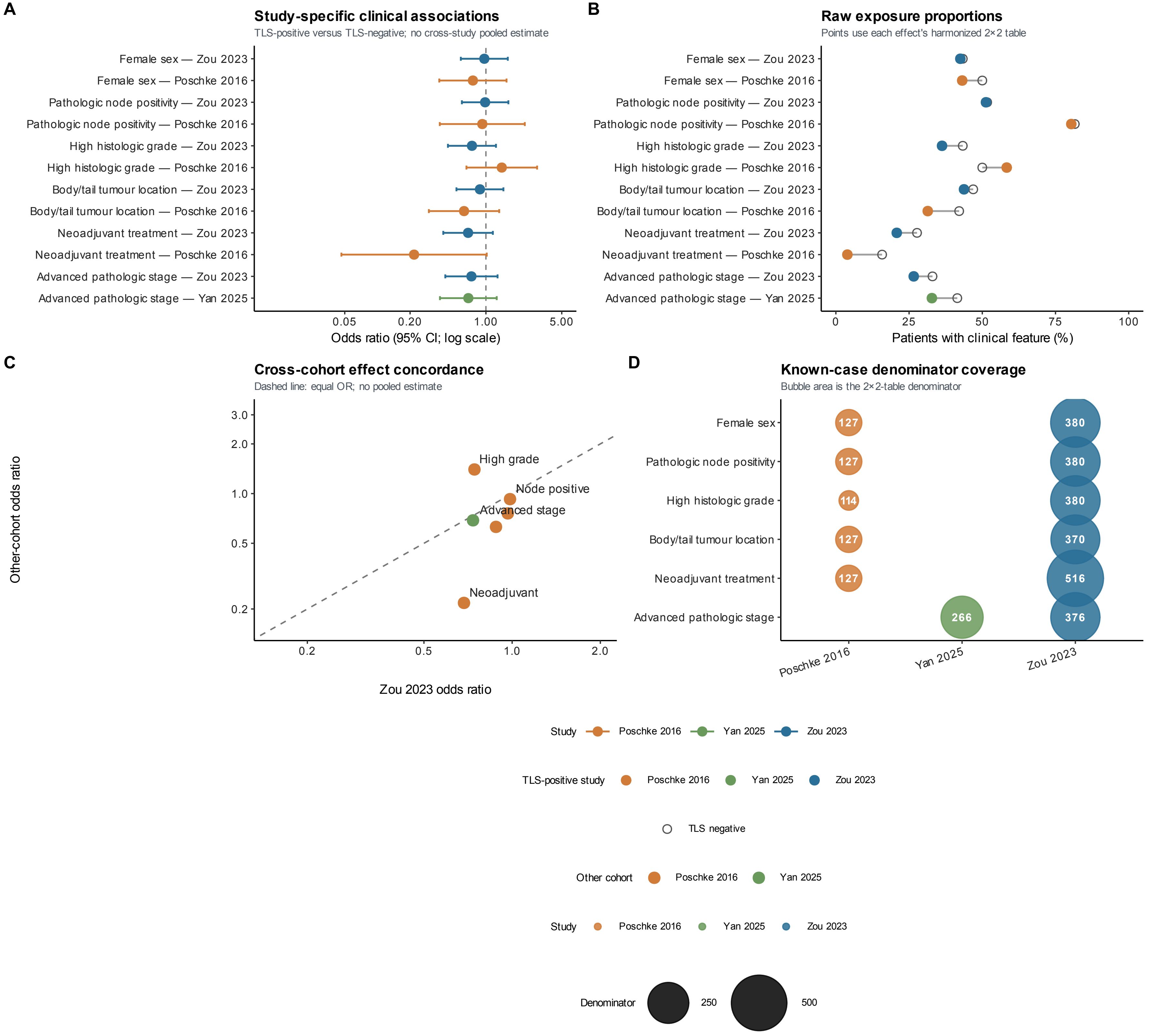

A
Study-specific clinical associations
TLS-positive versus TLS-negative; no cross-study pooled estimate
Female sex — Zou 2023
Female sex — Poschke 2016
Pathologic node positivity — Zou 2023
Pathologic node positivity — Poschke 2016
High histologic grade — Zou 2023
High histologic grade — Poschke 2016
Body/tail tumour location — Zou 2023
Body/tail tumour location — Poschke 2016
Neoadjuvant treatment — Zou 2023
Neoadjuvant treatment — Poschke 2016
Advanced pathologic stage — Zou 2023
Advanced pathologic stage — Yan 2025
0.05
0.20
1.00
5.00
Odds ratio (95% CI; log scale)
B
Raw exposure proportions
Points use each effect's harmonized 2×2 table
0
25
50
75
100
Patients with clinical feature (%)
C
Cross-cohort effect concordance
Dashed line: equal OR; no pooled estimate
High grade
Node positive
Advanced stage
Neoadjuvant
Other-cohort odds ratio
Zou 2023 odds ratio
D
Known-case denominator coverage
Bubble area is the 2×2-table denominator
Female sex
Pathologic node positivity
High histologic grade
Body/tail tumour location
Neoadjuvant treatment
Advanced pathologic stage
127
380
127
380
114
380
127
370
127
516
266
376
Poschke 2016
Yan 2025
Zou 2023
Study
TLS-positive study
TLS negative
Other cohort
Denominator
250
500
