# Supplementary material for: Prognostic and Spatial Organization of Tertiary Lymphoid Structures in Pancreatic Ductal Adenocarcinoma
Source: Biomedicines. 2026 Oct 9;14(10):2287. doi: 10.3390/biomedicines14102287 (PMC13701594; doi:10.3390/biomedicines14102287)
Supplement: Supplementary file 1 [file biomedicines-14-02287-s001.zip › 03_Figures/Figure_S3.pdf]

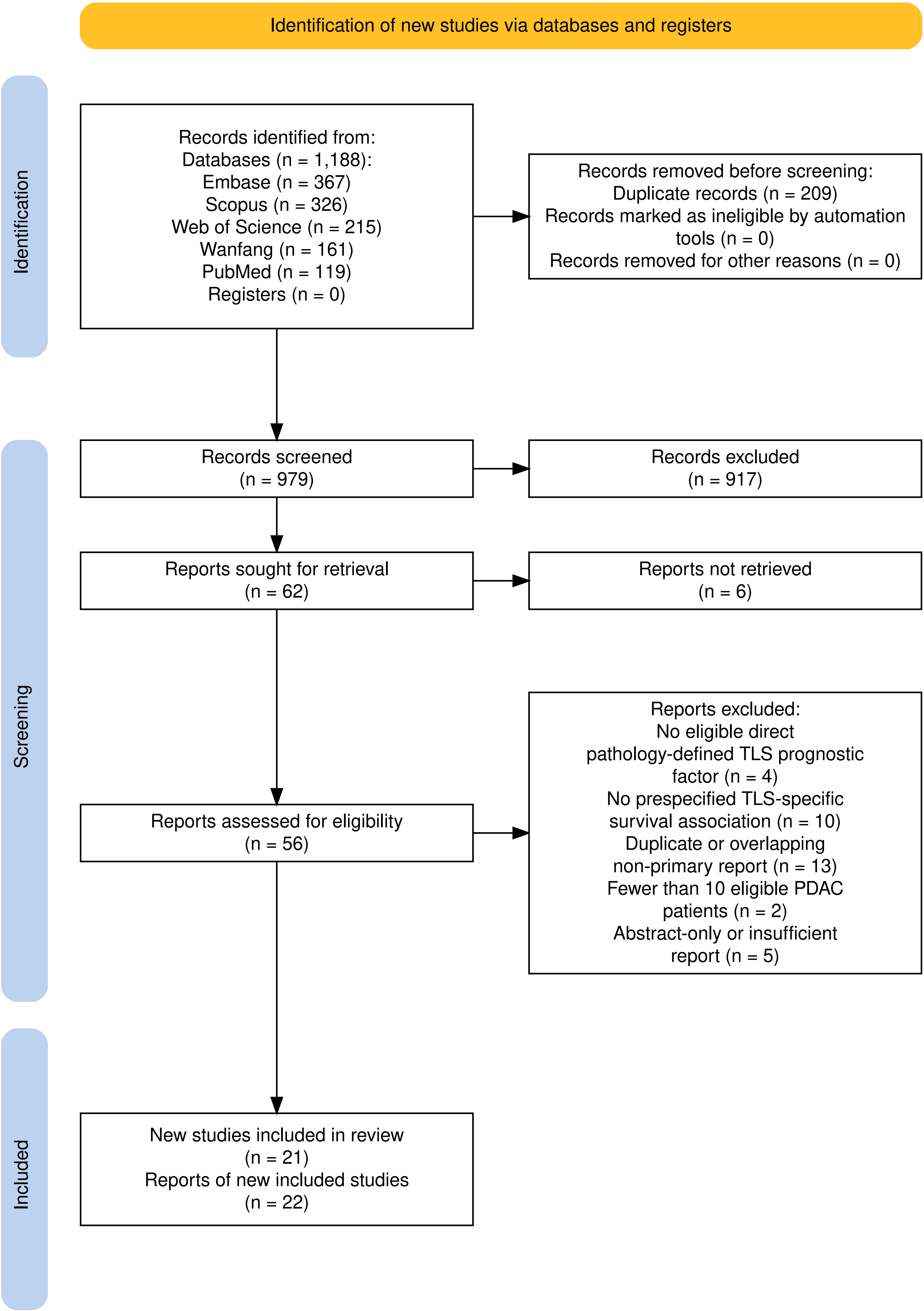
Identification of new studies via databases and registers
Identification
Screening
Included
Records identified from:
Databases (n = 1,188):
Embase (n = 367)
Scopus (n = 326)
Web of Science (n = 215)
Wanfang (n = 161)
PubMed (n = 119)
Registers (n = 0)
Records removed before screening:
Duplicate records (n = 209)
Records marked as ineligible by automation tools (n = 0)
Records removed for other reasons (n = 0)
Records screened (n = 979)
Records excluded (n = 917)
Reports sought for retrieval (n = 62)
Reports not retrieved (n = 6)
Reports assessed for eligibility (n = 56)
Reports excluded:
No eligible direct pathology-defined TLS prognostic factor (n = 4)
No prespecified TLS-specific survival association (n = 10)
Duplicate or overlapping non-primary report (n = 13)
Fewer than 10 eligible PDAC patients (n = 2)
Abstract-only or insufficient report (n = 5)
New studies included in review (n = 21)
Reports of new included studies (n = 22)
