# Supplementary material for: Prognostic and Spatial Organization of Tertiary Lymphoid Structures in Pancreatic Ductal Adenocarcinoma
Source: Biomedicines. 2026 Oct 9;14(10):2287. doi: 10.3390/biomedicines14102287 (PMC13701594; doi:10.3390/biomedicines14102287)
Supplement: Supplementary file 1 [file biomedicines-14-02287-s001.zip › 03_Figures/Figure_S4.pdf]

Supplementary Figure S4 | RCTD reference sensitivity and TLS-neighborhood analysis

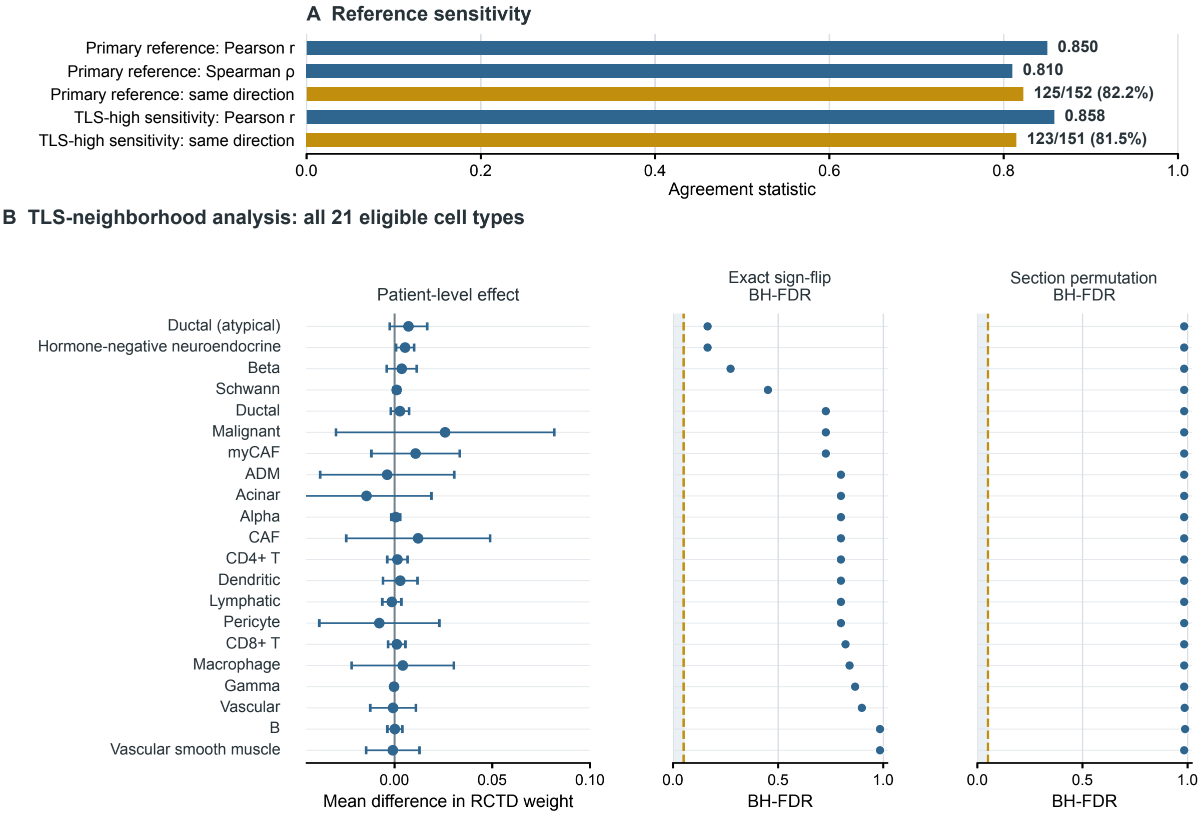

n=8 patients/sections; 999 permutations; image-pixel coordinates. Effect bars = patient mean ± SD. Dashed = q 0.05. Minimum q = 0.1641 / 0.9837; none < 0.05.
