# Supplementary material for: Prognostic and Spatial Organization of Tertiary Lymphoid Structures in Pancreatic Ductal Adenocarcinoma
Source: Biomedicines. 2026 Oct 9;14(10):2287. doi: 10.3390/biomedicines14102287 (PMC13701594; doi:10.3390/biomedicines14102287)
Supplement: Supplementary file 1 [file biomedicines-14-02287-s001.zip › 03_Figures/Figure_S5.pdf]

Figure S5 | Exploratory TLS-core distance gradients

A

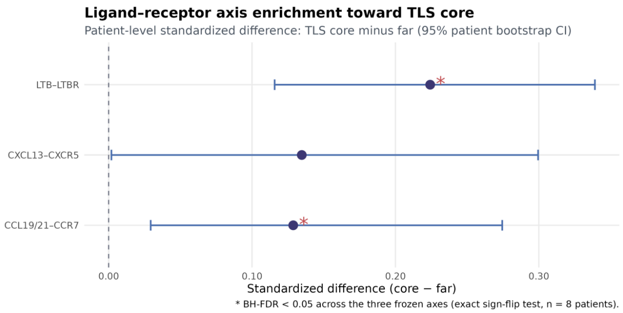

B

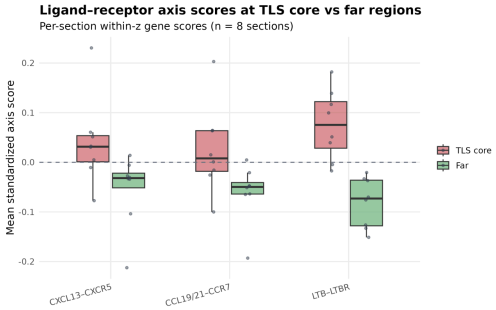

LTB–LTBR: mean core-minus-far standardized difference = 0.224; BH-FDR = 0.023; 8/8 positive

CCL19/CCL21–CCR7: mean core-minus-far standardized difference = 0.129; BH-FDR = 0.047; 7/8 positive

CXCL13–CXCR5: mean core-minus-far standardized difference = 0.135; BH-FDR = 0.125; 6/8 positive

S5A: patient-bootstrap 95% CI; S5B: 8 sections with per-section within-z scores
