# Supplementary material for: Prognostic and Spatial Organization of Tertiary Lymphoid Structures in Pancreatic Ductal Adenocarcinoma
Source: Biomedicines. 2026 Oct 9;14(10):2287. doi: 10.3390/biomedicines14102287 (PMC13701594; doi:10.3390/biomedicines14102287)
Supplement: Supplementary file 1 [file biomedicines-14-02287-s001.zip › prisma_2020_checklist_.pdf]

PRISMA 2020 Checklist

Manuscript: Prognostic and spatial organization of tertiary lymphoid structures in pancreatic ductal adenocarcinoma

Article This checklist records the reporting location for each PRISMA 2020 item.

Section and Topic	Item #	Checklist Item	Location Where Item Is Reported
TITLE	1	Identify the report as a systematic review.	Abstract and Methods identify the systematic-review component of this integrated Research Article; the article title reflects the full clinical-plus-molecular study design.
ABSTRACT	2	See the PRISMA 2020 for Abstracts checklist.	Abstract, Background/Objectives–Conclusions.
INTRODUCTION	3	Describe the rationale for the review in the context of existing knowledge.	Introduction.
INTRODUCTION	4	Provide an explicit statement of the objective(s) or question(s) addressed by the review.	Introduction; Materials and Methods.
METHODS	5	Specify the inclusion and exclusion criteria for the review and how studies were grouped for the syntheses.	Materials and Methods, "Systematic search and study selection".
METHODS	6	Specify all databases, registers, websites, organisations, reference lists and other sources searched or consulted. Specify the date when each source was last searched or consulted.	Materials and Methods, "Systematic search and study selection"; Supplementary Method S1; last search 25 July 2026.
METHODS	7	Present the full search strategies for all databases, registers and websites, including any filters and limits used.	Supplementary Method S1 reports the complete database-specific strategies, platforms, dates, limits, and yields.
METHODS	8	Specify the methods used to	Materials and

Section and Topic	Item #	Checklist Item	Location Where Item Is Reported
		decide whether a study met the inclusion criteria, including reviewer number and independence.	Methods, “Systematic search and study selection”: two independent reviewers; disagreements resolved by consensus and third-reviewer adjudication when required.
METHODS	9	Specify the methods used to collect data from reports, including reviewer number and independence.	Materials and Methods, “Clinical data extraction and effect harmonization”: duplicate independent extraction and source checking, with consensus/third-reviewer resolution.
METHODS	10a	List and define all outcomes for which data were sought.	Materials and Methods, “Eligible studies” and “Clinical data extraction and effect harmonization”.
METHODS	10b	List and define all other variables for which data were sought.	Materials and Methods, “Clinical data extraction and effect harmonization”.
METHODS	11	Specify the methods used to assess risk of bias in included studies.	Materials and Methods, “Risk of bias”; Figure S1.
METHODS	12	Specify the effect measure(s) used for each outcome.	Materials and Methods, “Clinical meta-analysis”.
METHODS	13a	Describe the processes used to decide which studies were eligible for each synthesis.	Materials and Methods, “Clinical data extraction and effect harmonization”.
METHODS	13b	Describe any methods required to prepare the data for presentation or synthesis.	Materials and Methods, “Clinical data extraction and effect harmonization”.
METHODS	13c	Describe methods used to tabulate or visually display results of individual studies and syntheses.	Results; Tables 1–2; Figures 1–9; Supplementary Figures S1–S3 and Tables S1–S3.
METHODS	13d	Describe methods used to	Materials and Methods,

Section and Topic	Item #	Checklist Item	Location Where Item Is Reported
		synthesize results and rationale for choices.	“Clinical meta-analysis”.
METHODS	13e	Describe methods used to explore causes of heterogeneity.	Materials and Methods, “Clinical meta-analysis”; Results, clinical heterogeneity analyses.
METHODS	13f	Describe sensitivity analyses conducted to assess robustness.	Materials and Methods, “Risk of bias” and “Clinical meta-analysis”; Results.
METHODS	14	Describe methods used to assess risk of bias due to missing results in a synthesis.	Limitations: formal small-study-effect tests were not conducted because each synthesis included fewer than ten studies.
METHODS	15	Describe methods used to assess certainty/confidence in the body of evidence.	Formal certainty-of-evidence assessment: not performed. Figure S1 reports QUIPS risk of bias.
RESULTS	16a	Describe the results of the search and selection process, from number of records identified to number of studies included.	Results, “Clinical evidence establishes a favorable but heterogeneous TLS phenotype”; Supplementary Figure S3.
RESULTS	16b	Cite studies that might appear to meet inclusion criteria but were excluded, and explain why.	Supplementary Figure S3 reports aggregate exclusion reasons; the screening ledger is retained in the review records.
RESULTS	17	Cite each included study and present its characteristics.	Table 1; Supplementary Table S1.
RESULTS	18	Present assessments of risk of bias for each included study.	Figure S1; Materials and Methods, “Risk of bias”.
RESULTS	19	For all outcomes, present for each study: summary statistics and an effect estimate with its precision.	Table 1; Figure 1. Individual study summary statistics and effect estimates are reported where available.
RESULTS	20a	For each synthesis, briefly summarise the characteristics and risk of bias among contributing studies.	Results, clinical evidence sections; Table 1; Figure S1.
RESULTS	20b	Present results of all statistical syntheses conducted.	Results; Figures 1–3.
RESULTS	20c	Present results of all investigations of possible causes of heterogeneity.	Results, TLS prevalence and clinical heterogeneity; Figures 1–3.
RESULTS	20d	Present results of all sensitivity analyses conducted.	Results, clinical risk-of-bias and leave-one-out sensitivity analyses; Figures 1–3.
RESULTS	21	Present assessments of risk of bias due to missing results for each synthesis	Limitations; formal small-study-effect tests not performed because k < 10.

Section and Topic	Item #	Checklist Item	Location Where Item Is Reported
		assessed.	
RESULTS	22	Present assessments of certainty/confidence in the body of evidence.	Formal certainty-of-evidence assessment: not performed.
DISCUSSION	23a	Provide a general interpretation of the results in the context of other evidence.	Discussion.
DISCUSSION	23b	Discuss limitations of the evidence included in the review.	Discussion; Limitations.
DISCUSSION	23c	Discuss limitations of the review processes used.	Limitations of the study, including late registration, absence of formal grey-literature searching, small synthesis sizes, and heterogeneous TLS definitions.
DISCUSSION	23d	Discuss implications of the results for practice, policy and future research.	Discussion.
OTHER	24a	Provide registration information for the review.	Materials and Methods: PROSPERO CRD420261465162.
OTHER	24b	Indicate where the review protocol can be accessed, or state that a protocol was not prepared.	PROSPERO record CRD420261465162; no separate pre-registration protocol was prepared.
OTHER	24c	Describe and explain any amendments to information provided at registration or in the protocol.	The registration occurred after screening and analysis initiation; this chronology is reported explicitly. No prospective-registration claim is made.
OTHER	25	Describe sources of financial or non-financial support and role of funders.	Funding statement.
OTHER	26	Declare competing interests of review authors.	Conflicts of Interest statement.
OTHER	27	Report which data, code and other materials are publicly available and where they can be found.	Availability of data and materials; Supplementary Code and Data

Section and Topic	Item #	Checklist Item	Location Where Item Is Reported
			supplied with the revision.

From: Page MJ, McKenzie JE, Bossuyt PM, et al. The PRISMA 2020 statement: an updated guideline for reporting systematic reviews. BMJ. 2021;372:n71. https://doi.org/10.1136/bmj.n71
